# Reconstructing evolutionary trajectories of mutation signature activities in cancer using TrackSig

**DOI:** 10.1038/s41467-020-14352-7

**Published:** 2020-02-05

**Authors:** Yulia Rubanova, Ruian Shi, Caitlin F. Harrigan, Roujia Li, Jeff Wintersinger, Nil Sahin, Amit G. Deshwar, PCAWG Evolution and Heterogeneity Working Group, Stefan C. Dentro, Ignaty Leshchiner, Moritz Gerstung, Clemency Jolly, Kerstin Haase, Maxime Tarabichi, Jeff Wintersinger, Amit G. Deshwar, Kaixian Yu, Santiago Gonzalez, Yulia Rubanova, Geoff Macintyre, David J. Adams, Pavana Anur, Rameen Beroukhim, Paul C. Boutros, David D. Bowtell, Peter J. Campbell, Shaolong Cao, Elizabeth L. Christie, Marek Cmero, Yupeng Cun, Kevin J. Dawson, Jonas Demeulemeester, Nilgun Donmez, Ruben M. Drews, Roland Eils, Yu Fan, Matthew Fittall, Dale W. Garsed, Gad Getz, Gavin Ha, Marcin Imielinski, Lara Jerman, Yuan Ji, Kortine Kleinheinz, Juhee Lee, Henry Lee-Six, Dimitri G. Livitz, Salem Malikic, Florian Markowetz, Inigo Martincorena, Thomas J. Mitchell, Ville Mustonen, Layla Oesper, Martin Peifer, Myron Peto, Benjamin J. Raphael, Daniel Rosebrock, S. Cenk Sahinalp, Adriana Salcedo, Matthias Schlesner, Steven Schumacher, Subhajit Sengupta, Ruian Shi, Seung Jun Shin, Oliver Spiro, Lincoln D. Stein, Ignacio Vázquez-García, Shankar Vembu, David A. Wheeler, Tsun-Po Yang, Xiaotong Yao, Ke Yuan, Hongtu Zhu, Wenyi Wang, Quaid D. Morris, Paul T. Spellman, David C. Wedge, Peter Van Loo, Quaid D. Morris, PCAWG Consortium, Lauri A. Aaltonen, Federico Abascal, Adam Abeshouse, Hiroyuki Aburatani, David J. Adams, Nishant Agrawal, Keun Soo Ahn, Sung-Min Ahn, Hiroshi Aikata, Rehan Akbani, Kadir C. Akdemir, Hikmat Al-Ahmadie, Sultan T. Al-Sedairy, Fatima Al-Shahrour, Malik Alawi, Monique Albert, Kenneth Aldape, Ludmil B. Alexandrov, Adrian Ally, Kathryn Alsop, Eva G. Alvarez, Fernanda Amary, Samirkumar B. Amin, Brice Aminou, Ole Ammerpohl, Matthew J. Anderson, Yeng Ang, Davide Antonello, Pavana Anur, Samuel Aparicio, Elizabeth L. Appelbaum, Yasuhito Arai, Axel Aretz, Koji Arihiro, Shun-ichi Ariizumi, Joshua Armenia, Laurent Arnould, Sylvia Asa, Yassen Assenov, Gurnit Atwal, Sietse Aukema, J. Todd Auman, Miriam R. R. Aure, Philip Awadalla, Marta Aymerich, Gary D. Bader, Adrian Baez-Ortega, Matthew H. Bailey, Peter J. Bailey, Miruna Balasundaram, Saianand Balu, Pratiti Bandopadhayay, Rosamonde E. Banks, Stefano Barbi, Andrew P. Barbour, Jonathan Barenboim, Jill Barnholtz-Sloan, Hugh Barr, Elisabet Barrera, John Bartlett, Javier Bartolome, Claudio Bassi, Oliver F. Bathe, Daniel Baumhoer, Prashant Bavi, Stephen B. Baylin, Wojciech Bazant, Duncan Beardsmore, Timothy A. Beck, Sam Behjati, Andreas Behren, Beifang Niu, Cindy Bell, Sergi Beltran, Christopher Benz, Andrew Berchuck, Anke K. Bergmann, Erik N. Bergstrom, Benjamin P. Berman, Daniel M. Berney, Stephan H. Bernhart, Rameen Beroukhim, Mario Berrios, Samantha Bersani, Johanna Bertl, Miguel Betancourt, Vinayak Bhandari, Shriram G. Bhosle, Andrew V. Biankin, Matthias Bieg, Darell Bigner, Hans Binder, Ewan Birney, Michael Birrer, Nidhan K. Biswas, Bodil Bjerkehagen, Tom Bodenheimer, Lori Boice, Giada Bonizzato, Johann S. De Bono, Arnoud Boot, Moiz S. Bootwalla, Ake Borg, Arndt Borkhardt, Keith A. Boroevich, Ivan Borozan, Christoph Borst, Marcus Bosenberg, Mattia Bosio, Jacqueline Boultwood, Guillaume Bourque, Paul C. Boutros, G. Steven Bova, David T. Bowen, Reanne Bowlby, David D. L. Bowtell, Sandrine Boyault, Rich Boyce, Jeffrey Boyd, Alvis Brazma, Paul Brennan, Daniel S. Brewer, Arie B. Brinkman, Robert G. Bristow, Russell R. Broaddus, Jane E. Brock, Malcolm Brock, Annegien Broeks, Angela N. Brooks, Denise Brooks, Benedikt Brors, Søren Brunak, Timothy J. C. Bruxner, Alicia L. Bruzos, Alex Buchanan, Ivo Buchhalter, Christiane Buchholz, Susan Bullman, Hazel Burke, Birgit Burkhardt, Kathleen H. Burns, John Busanovich, Carlos D. Bustamante, Adam P. Butler, Atul J. Butte, Niall J. Byrne, Anne-Lise Børresen-Dale, Samantha J. Caesar-Johnson, Andy Cafferkey, Declan Cahill, Claudia Calabrese, Carlos Caldas, Fabien Calvo, Niedzica Camacho, Peter J. Campbell, Elias Campo, Cinzia Cantù, Shaolong Cao, Thomas E. Carey, Joana Carlevaro-Fita, Rebecca Carlsen, Ivana Cataldo, Mario Cazzola, Jonathan Cebon, Robert Cerfolio, Dianne E. Chadwick, Dimple Chakravarty, Don Chalmers, Calvin Wing Yiu Chan, Kin Chan, Michelle Chan-Seng-Yue, Vishal S. Chandan, David K. Chang, Stephen J. Chanock, Lorraine A. Chantrill, Aurélien Chateigner, Nilanjan Chatterjee, Kazuaki Chayama, Hsiao-Wei Chen, Jieming Chen, Ken Chen, Yiwen Chen, Zhaohong Chen, Andrew D. Cherniack, Jeremy Chien, Yoke-Eng Chiew, Suet-Feung Chin, Juok Cho, Sunghoon Cho, Jung Kyoon Choi, Wan Choi, Christine Chomienne, Zechen Chong, Su Pin Choo, Angela Chou, Angelika N. Christ, Elizabeth L. Christie, Eric Chuah, Carrie Cibulskis, Kristian Cibulskis, Sara Cingarlini, Peter Clapham, Alexander Claviez, Sean Cleary, Nicole Cloonan, Marek Cmero, Colin C. Collins, Ashton A. Connor, Susanna L. Cooke, Colin S. Cooper, Leslie Cope, Vincenzo Corbo, Matthew G. Cordes, Stephen M. Cordner, Isidro Cortés-Ciriano, Kyle Covington, Prue A. Cowin, Brian Craft, David Craft, Chad J. Creighton, Yupeng Cun, Erin Curley, Ioana Cutcutache, Karolina Czajka, Bogdan Czerniak, Rebecca A. Dagg, Ludmila Danilova, Maria Vittoria Davi, Natalie R. Davidson, Helen Davies, Ian J. Davis, Brandi N. Davis-Dusenbery, Kevin J. Dawson, Francisco M. De La Vega, Ricardo De Paoli-Iseppi, Timothy Defreitas, Angelo P. Dei Tos, Olivier Delaneau, John A. Demchok, Jonas Demeulemeester, German M. Demidov, Deniz Demircioğlu, Nening M. Dennis, Robert E. Denroche, Stefan C. Dentro, Nikita Desai, Vikram Deshpande, Amit G. Deshwar, Christine Desmedt, Jordi Deu-Pons, Noreen Dhalla, Neesha C. Dhani, Priyanka Dhingra, Rajiv Dhir, Anthony DiBiase, Klev Diamanti, Li Ding, Shuai Ding, Huy Q. Dinh, Luc Dirix, HarshaVardhan Doddapaneni, Nilgun Donmez, Michelle T. Dow, Ronny Drapkin, Oliver Drechsel, Ruben M. Drews, Serge Serge, Tim Dudderidge, Ana Dueso-Barroso, Andrew J. Dunford, Michael Dunn, Lewis Jonathan Dursi, Fraser R. Duthie, Ken Dutton-Regester, Jenna Eagles, Douglas F. Easton, Stuart Edmonds, Paul A. Edwards, Sandra E. Edwards, Rosalind A. Eeles, Anna Ehinger, Juergen Eils, Roland Eils, Adel El-Naggar, Matthew Eldridge, Kyle Ellrott, Serap Erkek, Georgia Escaramis, Shadrielle M. G. Espiritu, Xavier Estivill, Dariush Etemadmoghadam, Jorunn E. Eyfjord, Bishoy M. Faltas, Daiming Fan, Yu Fan, William C. Faquin, Claudiu Farcas, Matteo Fassan, Aquila Fatima, Francesco Favero, Nodirjon Fayzullaev, Ina Felau, Sian Fereday, Martin L. Ferguson, Vincent Ferretti, Lars Feuerbach, Matthew A. Field, J. Lynn Fink, Gaetano Finocchiaro, Cyril Fisher, Matthew W. Fittall, Anna Fitzgerald, Rebecca C. Fitzgerald, Adrienne M. Flanagan, Neil E. Fleshner, Paul Flicek, John A. Foekens, Kwun M. Fong, Nuno A. Fonseca, Christopher S. Foster, Natalie S. Fox, Michael Fraser, Scott Frazer, Milana Frenkel-Morgenstern, William Friedman, Joan Frigola, Catrina C. Fronick, Akihiro Fujimoto, Masashi Fujita, Masashi Fukayama, Lucinda A. Fulton, Robert S. Fulton, Mayuko Furuta, P. Andrew Futreal, Anja Füllgrabe, Stacey B. Gabriel, Steven Gallinger, Carlo Gambacorti-Passerini, Jianjiong Gao, Shengjie Gao, Levi Garraway, Øystein Garred, Erik Garrison, Dale W. Garsed, Nils Gehlenborg, Josep L. L. Gelpi, Joshy George, Daniela S. Gerhard, Clarissa Gerhauser, Jeffrey E. Gershenwald, Mark Gerstein, Moritz Gerstung, Gad Getz, Mohammed Ghori, Ronald Ghossein, Nasra H. Giama, Richard A. Gibbs, Bob Gibson, Anthony J. Gill, Pelvender Gill, Dilip D. Giri, Dominik Glodzik, Vincent J. Gnanapragasam, Maria Elisabeth Goebler, Mary J. Goldman, Carmen Gomez, Santiago Gonzalez, Abel Gonzalez-Perez, Dmitry A. Gordenin, James Gossage, Kunihito Gotoh, Ramaswamy Govindan, Dorthe Grabau, Janet S. Graham, Robert C. Grant, Anthony R. Green, Eric Green, Liliana Greger, Nicola Grehan, Sonia Grimaldi, Sean M. Grimmond, Robert L. Grossman, Adam Grundhoff, Gunes Gundem, Qianyun Guo, Manaswi Gupta, Shailja Gupta, Ivo G. Gut, Marta Gut, Jonathan Göke, Gavin Ha, Andrea Haake, David Haan, Siegfried Haas, Kerstin Haase, James E. Haber, Nina Habermann, Faraz Hach, Syed Haider, Natsuko Hama, Freddie C. Hamdy, Anne Hamilton, Mark P. Hamilton, Leng Han, George B. Hanna, Martin Hansmann, Nicholas J. Haradhvala, Olivier Harismendy, Ivon Harliwong, Arif O. Harmanci, Eoghan Harrington, Takanori Hasegawa, David Haussler, Steve Hawkins, Shinya Hayami, Shuto Hayashi, D. Neil Hayes, Stephen J. Hayes, Nicholas K. Hayward, Steven Hazell, Yao He, Allison P. Heath, Simon C. Heath, David Hedley, Apurva M. Hegde, David I. Heiman, Michael C. Heinold, Zachary Heins, Lawrence E. Heisler, Eva Hellstrom-Lindberg, Mohamed Helmy, Seong Gu Heo, Austin J. Hepperla, José María Heredia-Genestar, Carl Herrmann, Peter Hersey, Julian M. Hess, Holmfridur Hilmarsdottir, Jonathan Hinton, Satoshi Hirano, Nobuyoshi Hiraoka, Katherine A. Hoadley, Asger Hobolth, Ermin Hodzic, Jessica I. Hoell, Steve Hoffmann, Oliver Hofmann, Andrea Holbrook, Aliaksei Z. Holik, Michael A. Hollingsworth, Oliver Holmes, Robert A. Holt, Chen Hong, Eun Pyo Hong, Jongwhi H. Hong, Gerrit K. Hooijer, Henrik Hornshøj, Fumie Hosoda, Yong Hou, Volker Hovestadt, William Howat, Alan P. Hoyle, Ralph H. Hruban, Jianhong Hu, Taobo Hu, Xing Hua, Kuan-lin Huang, Mei Huang, Mi Ni Huang, Vincent Huang, Yi Huang, Wolfgang Huber, Thomas J. Hudson, Michael Hummel, Jillian A. Hung, David Huntsman, Ted R. Hupp, Jason Huse, Matthew R. Huska, Barbara Hutter, Carolyn M. Hutter, Daniel Hübschmann, Christine A. Iacobuzio-Donahue, Charles David Imbusch, Marcin Imielinski, Seiya Imoto, William B. Isaacs, Keren Isaev, Shumpei Ishikawa, Murat Iskar, S. M. Ashiqul Islam, Michael Ittmann, Sinisa Ivkovic, Jose M. G. Izarzugaza, Jocelyne Jacquemier, Valerie Jakrot, Nigel B. Jamieson, Gun Ho Jang, Se Jin Jang, Joy C. Jayaseelan, Reyka Jayasinghe, Stuart R. Jefferys, Karine Jegalian, Jennifer L. Jennings, Seung-Hyup Jeon, Lara Jerman, Yuan Ji, Wei Jiao, Peter A. Johansson, Amber L. Johns, Jeremy Johns, Rory Johnson, Todd A. Johnson, Clemency Jolly, Yann Joly, Jon G. Jonasson, Corbin D. Jones, David R. Jones, David T. W. Jones, Nic Jones, Steven J. M. Jones, Jos Jonkers, Young Seok Ju, Hartmut Juhl, Jongsun Jung, Malene Juul, Randi Istrup Juul, Sissel Juul, Natalie Jäger, Rolf Kabbe, Andre Kahles, Abdullah Kahraman, Vera B. Kaiser, Hojabr Kakavand, Sangeetha Kalimuthu, Christof von Kalle, Koo Jeong Kang, Katalin Karaszi, Beth Karlan, Rosa Karlić, Dennis Karsch, Katayoon Kasaian, Karin S. Kassahn, Hitoshi Katai, Mamoru Kato, Hiroto Katoh, Yoshiiku Kawakami, Jonathan D. Kay, Stephen H. Kazakoff, Marat D. Kazanov, Maria Keays, Electron Kebebew, Richard F. Kefford, Manolis Kellis, James G. Kench, Catherine J. Kennedy, Jules N. A. Kerssemakers, David Khoo, Vincent Khoo, Narong Khuntikeo, Ekta Khurana, Helena Kilpinen, Hark Kyun Kim, Hyung-Lae Kim, Hyung-Yong Kim, Hyunghwan Kim, Jaegil Kim, Jihoon Kim, Jong K. Kim, Youngwook Kim, Tari A. King, Wolfram Klapper, Kortine Kleinheinz, Leszek J. Klimczak, Stian Knappskog, Michael Kneba, Bartha M. Knoppers, Youngil Koh, Jan Komorowski, Daisuke Komura, Mitsuhiro Komura, Gu Kong, Marcel Kool, Jan O. Korbel, Viktoriya Korchina, Andrey Korshunov, Michael Koscher, Roelof Koster, Zsofia Kote-Jarai, Antonios Koures, Milena Kovacevic, Barbara Kremeyer, Helene Kretzmer, Markus Kreuz, Savitri Krishnamurthy, Dieter Kube, Kiran Kumar, Pardeep Kumar, Sushant Kumar, Yogesh Kumar, Ritika Kundra, Kirsten Kübler, Ralf Küppers, Jesper Lagergren, Phillip H. Lai, Peter W. Laird, Sunil R. Lakhani, Christopher M. Lalansingh, Emilie Lalonde, Fabien C. Lamaze, Adam Lambert, Eric Lander, Pablo Landgraf, Luca Landoni, Anita Langerød, Andrés Lanzós, Denis Larsimont, Erik Larsson, Mark Lathrop, Loretta M. S. Lau, Chris Lawerenz, Rita T. Lawlor, Michael S. Lawrence, Alexander J. Lazar, Ana Mijalkovic Lazic, Xuan Le, Darlene Lee, Donghoon Lee, Eunjung Alice Lee, Hee Jin Lee, Jake June-Koo Lee, Jeong-Yeon Lee, Juhee Lee, Ming Ta Michael Lee, Henry Lee-Six, Kjong-Van Lehmann, Hans Lehrach, Dido Lenze, Conrad R. Leonard, Daniel A. Leongamornlert, Ignaty Leshchiner, Louis Letourneau, Ivica Letunic, Douglas A. Levine, Lora Lewis, Tim Ley, Chang Li, Constance H. Li, Haiyan Irene Li, Jun Li, Lin Li, Shantao Li, Siliang Li, Xiaobo Li, Xiaotong Li, Xinyue Li, Yilong Li, Han Liang, Sheng-Ben Liang, Peter Lichter, Pei Lin, Ziao Lin, W. M. Linehan, Ole Christian Lingjærde, Dongbing Liu, Eric Minwei Liu, Fei-Fei Fei Liu, Fenglin Liu, Jia Liu, Xingmin Liu, Julie Livingstone, Dimitri Livitz, Naomi Livni, Lucas Lochovsky, Markus Loeffler, Georgina V. Long, Armando Lopez-Guillermo, Shaoke Lou, David N. Louis, Laurence B. Lovat, Yiling Lu, Yong-Jie Lu, Youyong Lu, Claudio Luchini, Ilinca Lungu, Xuemei Luo, Hayley J. Luxton, Andy G. Lynch, Lisa Lype, Cristina López, Carlos López-Otín, Eric Z. Ma, Yussanne Ma, Gaetan MacGrogan, Shona MacRae, Geoff Macintyre, Tobias Madsen, Kazuhiro Maejima, Andrea Mafficini, Dennis T. Maglinte, Arindam Maitra, Partha P. Majumder, Luca Malcovati, Salem Malikic, Giuseppe Malleo, Graham J. Mann, Luisa Mantovani-Löffler, Kathleen Marchal, Giovanni Marchegiani, Elaine R. Mardis, Adam A. Margolin, Maximillian G. Marin, Florian Markowetz, Julia Markowski, Jeffrey Marks, Tomas Marques-Bonet, Marco A. Marra, Luke Marsden, John W. M. Martens, Sancha Martin, Jose I. Martin-Subero, Iñigo Martincorena, Alexander Martinez-Fundichely, Yosef E. Maruvka, R. Jay Mashl, Charlie E. Massie, Thomas J. Matthew, Lucy Matthews, Erik Mayer, Simon Mayes, Michael Mayo, Faridah Mbabaali, Karen McCune, Ultan McDermott, Patrick D. McGillivray, Michael D. McLellan, John D. McPherson, John R. McPherson, Treasa A. McPherson, Samuel R. Meier, Alice Meng, Shaowu Meng, Andrew Menzies, Neil D. Merrett, Sue Merson, Matthew Meyerson, William Meyerson, Piotr A. Mieczkowski, George L. Mihaiescu, Sanja Mijalkovic, Tom Mikkelsen, Michele Milella, Linda Mileshkin, Christopher A. Miller, David K. Miller, Jessica K. Miller, Gordon B. Mills, Ana Milovanovic, Sarah Minner, Marco Miotto, Gisela Mir Arnau, Lisa Mirabello, Chris Mitchell, Thomas J. Mitchell, Satoru Miyano, Naoki Miyoshi, Shinichi Mizuno, Fruzsina Molnár-Gábor, Malcolm J. Moore, Richard A. Moore, Sandro Morganella, Quaid D. Morris, Carl Morrison, Lisle E. Mose, Catherine D. Moser, Ferran Muiños, Loris Mularoni, Andrew J. Mungall, Karen Mungall, Elizabeth A. Musgrove, Ville Mustonen, David Mutch, Francesc Muyas, Donna M. Muzny, Alfonso Muñoz, Jerome Myers, Ola Myklebost, Peter Möller, Genta Nagae, Adnan M. Nagrial, Hardeep K. Nahal-Bose, Hitoshi Nakagama, Hidewaki Nakagawa, Hiromi Nakamura, Toru Nakamura, Kaoru Nakano, Tannistha Nandi, Jyoti Nangalia, Mia Nastic, Arcadi Navarro, Fabio C. P. Navarro, David E. Neal, Gerd Nettekoven, Felicity Newell, Steven J. Newhouse, Yulia Newton, Alvin Wei Tian Ng, Anthony Ng, Jonathan Nicholson, David Nicol, Yongzhan Nie, G. Petur Nielsen, Morten Muhlig Nielsen, Serena Nik-Zainal, Michael S. Noble, Katia Nones, Paul A. Northcott, Faiyaz Notta, Brian D. O’Connor, Peter O’Donnell, Maria O’Donovan, Sarah O’Meara, Brian Patrick O’Neill, J. Robert O’Neill, David Ocana, Angelica Ochoa, Layla Oesper, Christopher Ogden, Hideki Ohdan, Kazuhiro Ohi, Lucila Ohno-Machado, Karin A. Oien, Akinyemi I. Ojesina, Hidenori Ojima, Takuji Okusaka, Larsson Omberg, Choon Kiat Ong, Stephan Ossowski, German Ott, B. F. Francis Ouellette, Christine P’ng, Marta Paczkowska, Salvatore Paiella, Chawalit Pairojkul, Marina Pajic, Qiang Pan-Hammarström, Elli Papaemmanuil, Irene Papatheodorou, Nagarajan Paramasivam, Ji Wan Park, Joong-Won Park, Keunchil Park, Kiejung Park, Peter J. Park, Joel S. Parker, Simon L. Parsons, Harvey Pass, Danielle Pasternack, Alessandro Pastore, Ann-Marie Patch, Iris Pauporté, Antonio Pea, John V. Pearson, Chandra Sekhar Pedamallu, Jakob Skou Pedersen, Paolo Pederzoli, Martin Peifer, Nathan A. Pennell, Charles M. Perou, Marc D. Perry, Gloria M. Petersen, Myron Peto, Nicholas Petrelli, Robert Petryszak, Stefan M. Pfister, Mark Phillips, Oriol Pich, Hilda A. Pickett, Todd D. Pihl, Nischalan Pillay, Sarah Pinder, Mark Pinese, Andreia V. Pinho, Esa Pitkänen, Xavier Pivot, Elena Piñeiro-Yáñez, Laura Planko, Christoph Plass, Paz Polak, Tirso Pons, Irinel Popescu, Olga Potapova, Aparna Prasad, Shaun R. Preston, Manuel Prinz, Antonia L. Pritchard, Stephenie D. Prokopec, Elena Provenzano, Xose S. Puente, Sonia Puig, Montserrat Puiggròs, Sergio Pulido-Tamayo, Gulietta M. Pupo, Colin A. Purdie, Michael C. Quinn, Raquel Rabionet, Janet S. Rader, Bernhard Radlwimmer, Petar Radovic, Benjamin Raeder, Keiran M. Raine, Manasa Ramakrishna, Kamna Ramakrishnan, Suresh Ramalingam, Benjamin J. Raphael, W. Kimryn Rathmell, Tobias Rausch, Guido Reifenberger, Jüri Reimand, Jorge Reis-Filho, Victor Reuter, Iker Reyes-Salazar, Matthew A. Reyna, Sheila M. Reynolds, Esther Rheinbay, Yasser Riazalhosseini, Andrea L. Richardson, Julia Richter, Matthew Ringel, Markus Ringnér, Yasushi Rino, Karsten Rippe, Jeffrey Roach, Lewis R. Roberts, Nicola D. Roberts, Steven A. Roberts, A. Gordon Robertson, Alan J. Robertson, Javier Bartolomé Rodriguez, Bernardo Rodriguez-Martin, F. Germán Rodríguez-González, Michael H. A. Roehrl, Marius Rohde, Hirofumi Rokutan, Gilles Romieu, Ilse Rooman, Tom Roques, Daniel Rosebrock, Mara Rosenberg, Philip C. Rosenstiel, Andreas Rosenwald, Edward W. Rowe, Romina Royo, Steven G. Rozen, Yulia Rubanova, Mark A. Rubin, Carlota Rubio-Perez, Vasilisa A. Rudneva, Borislav C. Rusev, Andrea Ruzzenente, Gunnar Rätsch, Radhakrishnan Sabarinathan, Veronica Y. Sabelnykova, Sara Sadeghi, S. Cenk Sahinalp, Natalie Saini, Mihoko Saito-Adachi, Gordon Saksena, Adriana Salcedo, Roberto Salgado, Leonidas Salichos, Richard Sallari, Charles Saller, Roberto Salvia, Michelle Sam, Jaswinder S. Samra, Francisco Sanchez-Vega, Chris Sander, Grant Sanders, Rajiv Sarin, Iman Sarrafi, Aya Sasaki-Oku, Torill Sauer, Guido Sauter, Robyn P. M. Saw, Maria Scardoni, Christopher J. Scarlett, Aldo Scarpa, Ghislaine Scelo, Dirk Schadendorf, Jacqueline E. Schein, Markus B. Schilhabel, Matthias Schlesner, Thorsten Schlomm, Heather K. Schmidt, Sarah-Jane Schramm, Stefan Schreiber, Nikolaus Schultz, Steven E. Schumacher, Roland F. Schwarz, Richard A. Scolyer, David Scott, Ralph Scully, Raja Seethala, Ayellet V. Segre, Iris Selander, Colin A. Semple, Yasin Senbabaoglu, Subhajit Sengupta, Elisabetta Sereni, Stefano Serra, Dennis C. Sgroi, Mark Shackleton, Nimish C. Shah, Sagedeh Shahabi, Catherine A. Shang, Ping Shang, Ofer Shapira, Troy Shelton, Ciyue Shen, Hui Shen, Rebecca Shepherd, Ruian Shi, Yan Shi, Yu-Jia Shiah, Tatsuhiro Shibata, Juliann Shih, Eigo Shimizu, Kiyo Shimizu, Seung Jun Shin, Yuichi Shiraishi, Tal Shmaya, Ilya Shmulevich, Solomon I. Shorser, Charles Short, Raunak Shrestha, Suyash S. Shringarpure, Craig Shriver, Shimin Shuai, Nikos Sidiropoulos, Reiner Siebert, Anieta M. Sieuwerts, Lina Sieverling, Sabina Signoretti, Katarzyna O. Sikora, Michele Simbolo, Ronald Simon, Janae V. Simons, Jared T. Simpson, Peter T. Simpson, Samuel Singer, Nasa Sinnott-Armstrong, Payal Sipahimalani, Tara J. Skelly, Marcel Smid, Jaclyn Smith, Karen Smith-McCune, Nicholas D. Socci, Heidi J. Sofia, Matthew G. Soloway, Lei Song, Anil K. Sood, Sharmila Sothi, Christos Sotiriou, Cameron M. Soulette, Paul N. Span, Paul T. Spellman, Nicola Sperandio, Andrew J. Spillane, Oliver Spiro, Jonathan Spring, Johan Staaf, Peter F. Stadler, Peter Staib, Stefan G. Stark, Lucy Stebbings, Ólafur Andri Stefánsson, Oliver Stegle, Lincoln D. Stein, Alasdair Stenhouse, Chip Stewart, Stephan Stilgenbauer, Miranda D. Stobbe, Michael R. Stratton, Jonathan R. Stretch, Adam J. Struck, Joshua M. Stuart, Henk G. Stunnenberg, Hong Su, Xiaoping Su, Ren X. Sun, Stephanie Sungalee, Hana Susak, Akihiro Suzuki, Fred Sweep, Monika Szczepanowski, Holger Sültmann, Takashi Yugawa, Angela Tam, David Tamborero, Benita Kiat Tee Tan, Donghui Tan, Patrick Tan, Hiroko Tanaka, Hirokazu Taniguchi, Tomas J. Tanskanen, Maxime Tarabichi, Roy Tarnuzzer, Patrick Tarpey, Morgan L. Taschuk, Kenji Tatsuno, Simon Tavaré, Darrin F. Taylor, Amaro Taylor-Weiner, Jon W. Teague, Bin Tean Teh, Varsha Tembe, Javier Temes, Kevin Thai, Sarah P. Thayer, Nina Thiessen, Gilles Thomas, Sarah Thomas, Alan Thompson, Alastair M. Thompson, John F. F. Thompson, R. Houston Thompson, Heather Thorne, Leigh B. Thorne, Adrian Thorogood, Grace Tiao, Nebojsa Tijanic, Lee E. Timms, Roberto Tirabosco, Marta Tojo, Stefania Tommasi, Christopher W. Toon, Umut H. Toprak, David Torrents, Giampaolo Tortora, Jörg Tost, Yasushi Totoki, David Townend, Nadia Traficante, Isabelle Treilleux, Jean-Rémi Trotta, Lorenz H. P. Trümper, Ming Tsao, Tatsuhiko Tsunoda, Jose M. C. Tubio, Olga Tucker, Richard Turkington, Daniel J. Turner, Andrew Tutt, Masaki Ueno, Naoto T. Ueno, Christopher Umbricht, Husen M. Umer, Timothy J. Underwood, Lara Urban, Tomoko Urushidate, Tetsuo Ushiku, Liis Uusküla-Reimand, Alfonso Valencia, David J. Van Den Berg, Steven Van Laere, Peter Van Loo, Erwin G. Van Meir, Gert G. Van den Eynden, Theodorus Van der Kwast, Naveen Vasudev, Miguel Vazquez, Ravikiran Vedururu, Umadevi Veluvolu, Shankar Vembu, Lieven P. C. Verbeke, Peter Vermeulen, Clare Verrill, Alain Viari, David Vicente, Caterina Vicentini, K. VijayRaghavan, Juris Viksna, Ricardo E. Vilain, Izar Villasante, Anne Vincent-Salomon, Tapio Visakorpi, Douglas Voet, Paresh Vyas, Ignacio Vázquez-García, Nick M. Waddell, Nicola Waddell, Claes Wadelius, Lina Wadi, Rabea Wagener, Jeremiah A. Wala, Jian Wang, Jiayin Wang, Linghua Wang, Qi Wang, Wenyi Wang, Yumeng Wang, Zhining Wang, Paul M. Waring, Hans-Jörg Warnatz, Jonathan Warrell, Anne Y. Warren, Sebastian M. Waszak, David C. Wedge, Dieter Weichenhan, Paul Weinberger, John N. Weinstein, Joachim Weischenfeldt, Daniel J. Weisenberger, Ian Welch, Michael C. Wendl, Johannes Werner, Justin P. Whalley, David A. Wheeler, Hayley C. Whitaker, Dennis Wigle, Matthew D. Wilkerson, Ashley Williams, James S. Wilmott, Gavin W. Wilson, Julie M. Wilson, Richard K. Wilson, Boris Winterhoff, Jeffrey A. Wintersinger, Maciej Wiznerowicz, Stephan Wolf, Bernice H. Wong, Tina Wong, Winghing Wong, Youngchoon Woo, Scott Wood, Bradly G. Wouters, Adam J. Wright, Derek W. Wright, Mark H. Wright, Chin-Lee Wu, Dai-Ying Wu, Guanming Wu, Jianmin Wu, Kui Wu, Yang Wu, Zhenggang Wu, Liu Xi, Tian Xia, Qian Xiang, Xiao Xiao, Rui Xing, Heng Xiong, Qinying Xu, Yanxun Xu, Hong Xue, Shinichi Yachida, Sergei Yakneen, Rui Yamaguchi, Takafumi N. Yamaguchi, Masakazu Yamamoto, Shogo Yamamoto, Hiroki Yamaue, Fan Yang, Huanming Yang, Jean Y. Yang, Liming Yang, Lixing Yang, Shanlin Yang, Tsun-Po Yang, Yang Yang, Xiaotong Yao, Marie-Laure Yaspo, Lucy Yates, Christina Yau, Chen Ye, Kai Ye, Venkata D. Yellapantula, Christopher J. Yoon, Sung-Soo Yoon, Fouad Yousif, Jun Yu, Kaixian Yu, Willie Yu, Yingyan Yu, Ke Yuan, Yuan Yuan, Denis Yuen, Christina K. Yung, Olga Zaikova, Jorge Zamora, Marc Zapatka, Jean C. Zenklusen, Thorsten Zenz, Nikolajs Zeps, Cheng-Zhong Zhang, Fan Zhang, Hailei Zhang, Hongwei Zhang, Hongxin Zhang, Jiashan Zhang, Jing Zhang, Junjun Zhang, Xiuqing Zhang, Xuanping Zhang, Yan Zhang, Zemin Zhang, Zhongming Zhao, Liangtao Zheng, Xiuqing Zheng, Wanding Zhou, Yong Zhou, Bin Zhu, Hongtu Zhu, Jingchun Zhu, Shida Zhu, Lihua Zou, Xueqing Zou, Anna deFazio, Nicholas van As, Carolien H. M. van Deurzen, Marc J. van de Vijver, L. van’t Veer, Christian von Mering

**Affiliations:** 1 Department of Computer Science, University of Toronto, Toronto, ON Canada; 2 Vector Institute, Toronto, ON Canada; 3 University of Toronto, Toronto, ON Canada; 4 Donnelly Centre for Cellular and Biomolecular Research, University of Toronto, Toronto, ON Canada; 5 Department of Molecular Genetics, University of Toronto, Toronto, ON M5S 1A8 Canada; 6 The Edward S. Rogers Sr. Department of Electrical and Computer Engineering, University of Toronto, Toronto, ON Canada; 7 The Francis Crick Institute, London, NW1 1AT UK; 8 Wellcome Trust Sanger Institute, Cambridge, CB10 1SA UK; 9 Big Data Institute, University of Oxford, Oxford, OX3 7LF UK; 10 Broad Institute of MIT and Harvard, Cambridge, MA 02142 USA; 11 European Molecular Biology Laboratory, European Bioinformatics Institute (EMBL-EBI), Cambridge, CB10 1SD UK; 12 University of Toronto, Toronto, ON M5S 3E1 Canada; 13 The University of Texas MD Anderson Cancer Center, Houston, TX 77030 USA; 14 Cancer Research UK Cambridge Institute, University of Cambridge, Cambridge, CB2 0RE UK; 15 Molecular and Medical Genetics, Oregon Health and Science University, Portland, OR 97231 USA; 16 Dana-Farber Cancer Institute, Boston, MA 02215 USA; 17 Ontario Institute for Cancer Research, Toronto, ON M5G 0A3 Canada; 18 University of California, Los Angeles, CA 90095 USA; 19 Peter MacCallum Cancer Centre, Melbourne, VIC 3000 Australia; 20 University of Melbourne, Melbourne, VIC 3010 Australia; 21 Walter and Eliza Hall Institute, Melbourne, VIC 3000 Australia; 22 University of Cologne, 50931 Cologne, Germany; 23 University of Leuven, B-3000 Leuven, Belgium; 24 Simon Fraser University, Burnaby, BC V5A 1S6 Canada; 25 Vancouver Prostate Centre, Vancouver, BC V6H 3Z6 Canada; 26 German Cancer Research Center (DKFZ), 69120 Heidelberg, Germany; 27 Heidelberg University, 69120 Heidelberg, Germany; 28 Massachusetts General Hospital Center for Cancer Research, Charlestown, MA 02129 USA; 29 Department of Pathology, Massachusetts General Hospital, Boston, MA 02114 USA; 30 Harvard Medical School, Boston, MA 02215 USA; 31 Weill Cornell Medicine, New York, NY 10065 USA; 32 New York Genome Center, New York, NY 10013 USA; 33 University of Ljubljana, 1000 Ljubljana, Slovenia; 34 NorthShore University HealthSystem, Evanston, IL 60201 USA; 35 The University of Chicago, Chicago, IL 60637 USA; 36 University of California Santa Cruz, Santa Cruz, CA 95064 USA; 37 University of Cambridge, Cambridge, CB2 0QQ UK; 38 University of Helsinki, 00014 Helsinki, Finland; 39 Carleton College, Northfield, MN 55057 USA; 40 Princeton University, Princeton, NJ 08540 USA; 41 Indiana University, Bloomington, IN 47405 USA; 42 Korea University, Seoul, 02481 Republic of Korea; 43 Human Genome Sequencing Center, Baylor College of Medicine, Houston, TX 77030 USA; 44 University of Glasgow, Glasgow, G12 8RZ UK; 45 Oxford NIHR Biomedical Research Centre, Oxford, OX4 2PG UK; 200 Applied Tumor Genomics Research Program, Research Programs Unit, University of Helsinki, Helsinki, Finland; 201 Wellcome Sanger Institute, Wellcome Genome Campus, Hinxton, UK; 202 Memorial Sloan Kettering Cancer Center, New York, NY USA; 203 Genome Science Division, Research Center for Advanced Science and Technology, University of Tokyo, Tokyo, Japan; 204 Department of Surgery, University of Chicago, Chicago, IL USA; 205 Department of Surgery, Division of Hepatobiliary and Pancreatic Surgery, School of Medicine, Keimyung University Dongsan Medical Center, Daegu, South Korea; 206 Department of Oncology, Gil Medical Center, Gachon University, Incheon, South Korea; 207 Hiroshima University, Hiroshima, Japan; 208 Department of Bioinformatics and Computational Biology, The University of Texas MD Anderson Cancer Center, Houston, TX USA; 209 University of Texas MD Anderson Cancer Center, Houston, TX USA; 210 King Faisal Specialist Hospital and Research Centre, Al Maather, Riyadh, Saudi Arabia; 211 Bioinformatics Unit, Spanish National Cancer Research Centre (CNIO), Madrid, Spain; 212 Bioinformatics Core Facility, University Medical Center Hamburg, Hamburg, Germany; 213 Heinrich Pette Institute, Leibniz Institute for Experimental Virology, Hamburg, Germany; 214 Ontario Tumour Bank, Ontario Institute for Cancer Research, Toronto, ON Canada; 215 Department of Pathology, The University of Texas MD Anderson Cancer Center, Houston, TX USA; 216 Laboratory of Pathology, Center for Cancer Research, National Cancer Institute, Bethesda, MD USA; 217 Department of Cellular and Molecular Medicine and Department of Bioengineering, University of California San Diego, La Jolla, CA USA; 218 UC San Diego Moores Cancer Center, San Diego, CA USA; 219 Canada’s Michael Smith Genome Sciences Centre, BC Cancer, Vancouver, BC Canada; 220 Sir Peter MacCallum Department of Oncology, Peter MacCallum Cancer Centre, University of Melbourne, Melbourne, VIC Australia; 221 Centre for Research in Molecular Medicine and Chronic Diseases (CiMUS), Universidade de Santiago de Compostela, Santiago de Compostela, Spain; 222 Department of Zoology, Genetics and Physical Anthropology, (CiMUS), Universidade de Santiago de Compostela, Santiago de Compostela, Spain; 223 The Biomedical Research Centre (CINBIO), Universidade de Vigo, Vigo, Spain; 224 Royal National Orthopaedic Hospital - Bolsover, London, UK; 225 Department of Genomic Medicine, The University of Texas MD Anderson Cancer Center, Houston, TX USA; 226 Quantitative and Computational Biosciences Graduate Program, Baylor College of Medicine, Houston, TX USA; 227 The Jackson Laboratory for Genomic Medicine, Farmington, CT USA; 228 Genome Informatics Program, Ontario Institute for Cancer Research, Toronto, ON Canada; 229 Institute of Human Genetics, Christian-Albrechts-University, Kiel, Germany; 230 Institute of Human Genetics, Ulm University and Ulm University Medical Center, Ulm, Germany; 231 Queensland Centre for Medical Genomics, Institute for Molecular Bioscience, University of Queensland, St. Lucia, Brisbane, QLD Australia; 232 Salford Royal NHS Foundation Trust, Salford, UK; 233 Department of Surgery, Pancreas Institute, University and Hospital Trust of Verona, Verona, Italy; 234 Molecular and Medical Genetics, OHSU Knight Cancer Institute, Oregon Health and Science University, Portland, OR USA; 235 Department of Molecular Oncology, BC Cancer Research Centre, Vancouver, BC Canada; 236 The McDonnell Genome Institute at Washington University, St. Louis, MO USA; 237 University College London, London, UK; 238 Division of Cancer Genomics, National Cancer Center Research Institute, National Cancer Center, Tokyo, Japan; 239 DLR Project Management Agency, Bonn, Germany; 240 Tokyo Women’s Medical University, Tokyo, Japan; 241 Center for Molecular Oncology, Memorial Sloan Kettering Cancer Center, New York, NY USA; 242 Los Alamos National Laboratory, Los Alamos, NM USA; 243 Department of Pathology, University Health Network, Toronto General Hospital, Toronto, ON Canada; 244 Nottingham University Hospitals NHS Trust, Nottingham, UK; 245 Epigenomics and Cancer Risk Factors, German Cancer Research Center (DKFZ), Heidelberg, Germany; 246 Computational Biology Program, Ontario Institute for Cancer Research, Toronto, ON Canada; 247 Department of Molecular Genetics, University of Toronto, Toronto, ON Canada; 248 Vector Institute, Toronto, ON Canada; 249 Hematopathology Section, Institute of Pathology, Christian-Albrechts-University, Kiel, Germany; 250 Department of Pathology and Laboratory Medicine, School of Medicine, University of North Carolina at Chapel Hill, Chapel Hill, NC USA; 251 Department of Cancer Genetics, Institute for Cancer Research, Oslo University Hospital, The Norwegian Radium Hospital, Oslo, Norway; 252 Pathology, Hospital Clinic, Institut d’Investigacions Biomèdiques August Pi i Sunyer (IDIBAPS), University of Barcelona, Barcelona, Spain; 253 Department of Veterinary Medicine, Transmissible Cancer Group, University of Cambridge, Cambridge, UK; 254 Alvin J. Siteman Cancer Center, Washington University School of Medicine, St. Louis, MO USA; 255 Wolfson Wohl Cancer Research Centre, Institute of Cancer Sciences, University of Glasgow, Glasgow, UK; 256 Lineberger Comprehensive Cancer Center, University of North Carolina at Chapel Hill, Chapel Hill, NC USA; 257 Broad Institute of MIT and Harvard, Cambridge, MA USA; 258 Dana-Farber/Boston Children’s Cancer and Blood Disorders Center, Boston, MA USA; 259 Department of Pediatrics, Harvard Medical School, Boston, MA USA; 260 Leeds Institute of Medical Research @ St. James’s, University of Leeds, St. James’s University Hospital, Leeds, UK; 261 Department of Pathology and Diagnostics, University and Hospital Trust of Verona, Verona, Italy; 262 Department of Surgery, Princess Alexandra Hospital, Brisbane, QLD Australia; 263 Surgical Oncology Group, Diamantina Institute, University of Queensland, Brisbane, QLD Australia; 264 Department of Population and Quantitative Health Sciences, Case Western Reserve University School of Medicine, Cleveland, OH USA; 265 Research Health Analytics and Informatics, University Hospitals Cleveland Medical Center, Cleveland, OH USA; 266 Gloucester Royal Hospital, Gloucester, UK; 267 European Molecular Biology Laboratory, European Bioinformatics Institute (EMBL-EBI), Cambridge, UK; 268 Diagnostic Development, Ontario Institute for Cancer Research, Toronto, ON Canada; 269 Barcelona Supercomputing Center (BSC), Barcelona, Spain; 270 Arnie Charbonneau Cancer Institute, University of Calgary, Calgary, AB Canada; 271 Departments of Surgery and Oncology, University of Calgary, Calgary, AB Canada; 272 Department of Pathology, Oslo University Hospital, The Norwegian Radium Hospital, Oslo, Norway; 273 PanCuRx Translational Research Initiative, Ontario Institute for Cancer Research, Toronto, ON Canada; 274 Department of Oncology, Sidney Kimmel Comprehensive Cancer Center at Johns Hopkins University School of Medicine, Baltimore, MD USA; 275 University Hospital Southampton NHS Foundation Trust, Southampton, UK; 276 Royal Stoke University Hospital, Stoke-on-Trent, UK; 277 Genome Sequence Informatics, Ontario Institute for Cancer Research, Toronto, ON Canada; 278 Human Longevity Inc, San Diego, CA USA; 279 Olivia Newton-John Cancer Research Institute, La Trobe University, Heidelberg, VIC Australia; 280 Computer Network Information Center, Chinese Academy of Sciences, Beijing, China; 281 Genome Canada, Ottawa, ON Canada; 282 CNAG-CRG, Centre for Genomic Regulation (CRG), Barcelona Institute of Science and Technology (BIST), Barcelona, Spain; 283 Universitat Pompeu Fabra (UPF), Barcelona, Spain; 284 Buck Institute for Research on Aging, Novato, CA USA; 285 Duke University Medical Center, Durham, NC USA; 286 Department of Human Genetics, Hannover Medical School, Hannover, Germany; 287 Center for Bioinformatics and Functional Genomics, Cedars-Sinai Medical Center, Los Angeles, CA USA; 288 Department of Biomedical Sciences, Cedars-Sinai Medical Center, Los Angeles, CA USA; 289 The Hebrew University Faculty of Medicine, Jerusalem, Israel; 290 Barts Cancer Institute, Barts and the London School of Medicine and Dentistry, Queen Mary University of London, London, UK; 291 Department of Computer Science, Bioinformatics Group, University of Leipzig, Leipzig, Germany; 292 Interdisciplinary Center for Bioinformatics, University of Leipzig, Leipzig, Germany; 293 Transcriptome Bioinformatics, LIFE Research Center for Civilization Diseases, University of Leipzig, Leipzig, Germany; 294 Department of Medical Oncology, Dana-Farber Cancer Institute, Boston, MA USA; 295 Department of Cancer Biology, Dana-Farber Cancer Institute, Boston, MA USA; 296 Harvard Medical School, Boston, MA USA; 297 USC Norris Comprehensive Cancer Center, University of Southern California, Los Angeles, CA USA; 298 Department of Diagnostics and Public Health, University and Hospital Trust of Verona, Verona, Italy; 299 Department of Mathematics, Aarhus University, Aarhus, Denmark; 300 Department of Molecular Medicine (MOMA), Aarhus University Hospital, Aarhus N, Denmark; 301 Instituto Carlos Slim de la Salud, Mexico City, Mexico; 302 Department of Medical Biophysics, University of Toronto, Toronto, ON Canada; 303 Cancer Division, Garvan Institute of Medical Research, Kinghorn Cancer Centre, University of New South Wales (UNSW Sydney), Sydney, NSW Australia; 304 South Western Sydney Clinical School, Faculty of Medicine, University of New South Wales (UNSW Sydney), Liverpool, NSW Australia; 305 West of Scotland Pancreatic Unit, Glasgow Royal Infirmary, Glasgow, UK; 306 Center for Digital Health, Berlin Institute of Health and Charitè - Universitätsmedizin Berlin, Berlin, Germany; 307 Heidelberg Center for Personalized Oncology (DKFZ-HIPO), German Cancer Research Center (DKFZ), Heidelberg, Germany; 308 The Preston Robert Tisch Brain Tumor Center, Duke University Medical Center, Durham, NC USA; 309 Massachusetts General Hospital, Boston, MA USA; 310 National Institute of Biomedical Genomics, Kalyani, West Bengal India; 311 Institute of Clinical Medicine and Institute of Oral Biology, University of Oslo, Oslo, Norway; 312 University of North Carolina at Chapel Hill, Chapel Hill, NC USA; 313 ARC-Net Centre for Applied Research on Cancer, University and Hospital Trust of Verona, Verona, Italy; 314 The Institute of Cancer Research, London, UK; 315 Centre for Computational Biology, Duke-NUS Medical School, Singapore, Singapore; 316 Programme in Cancer and Stem Cell Biology, Duke-NUS Medical School, Singapore, Singapore; 317 Division of Oncology and Pathology, Department of Clinical Sciences Lund, Lund University, Lund, Sweden; 318 Department of Pediatric Oncology, Hematology and Clinical Immunology, Heinrich-Heine-University, Düsseldorf, Germany; 319 Laboratory for Medical Science Mathematics, RIKEN Center for Integrative Medical Sciences, Yokohama, Japan; 320 RIKEN Center for Integrative Medical Sciences, Yokohama, Japan; 321 Department of Internal Medicine/Hematology, Friedrich-Ebert-Hospital, Neumünster, Germany; 322 Departments of Dermatology and Pathology, Yale University, New Haven, CT USA; 323 Centre for Genomic Regulation (CRG), The Barcelona Institute of Science and Technology, Barcelona, Spain; 324 Radcliffe Department of Medicine, University of Oxford, Oxford, UK; 325 Canadian Center for Computational Genomics, McGill University, Montreal, QC Canada; 326 Department of Human Genetics, McGill University, Montreal, QC Canada; 327 Department of Human Genetics, University of California Los Angeles, Los Angeles, CA USA; 328 Department of Pharmacology, University of Toronto, Toronto, ON Canada; 329 Faculty of Medicine and Health Technology, Tampere University and Tays Cancer Center, Tampere University Hospital, Tampere, Finland; 330 Haematology, Leeds Teaching Hospitals NHS Trust, Leeds, UK; 331 Translational Research and Innovation, Centre Léon Bérard, Lyon, France; 332 Fox Chase Cancer Center, Philadelphia, PA USA; 333 International Agency for Research on Cancer, World Health Organization, Lyon, France; 334 Earlham Institute, Norwich, UK; 335 Norwich Medical School, University of East Anglia, Norwich, UK; 336 Department of Molecular Biology, Faculty of Science, Radboud Institute for Molecular Life Sciences, Radboud University, Nijmegen, HB The Netherlands; 337 CRUK Manchester Institute and Centre, Manchester, UK; 338 Department of Radiation Oncology, University of Toronto, Toronto, ON Canada; 339 Division of Cancer Sciences, Manchester Cancer Research Centre, University of Manchester, Manchester, UK; 340 Radiation Medicine Program, Princess Margaret Cancer Centre, Toronto, ON Canada; 341 Department of Pathology, Brigham and Women’s Hospital, Harvard Medical School, Boston, MA USA; 342 Department of Surgery, Division of Thoracic Surgery, The Johns Hopkins University School of Medicine, Baltimore, MD USA; 343 Division of Molecular Pathology, The Netherlands Cancer Institute, Oncode Institute, Amsterdam, CX The Netherlands; 344 Department of Biomolecular Engineering, University of California Santa Cruz, Santa Cruz, CA USA; 345 UC Santa Cruz Genomics Institute, University of California Santa Cruz, Santa Cruz, CA USA; 346 Division of Applied Bioinformatics, German Cancer Research Center (DKFZ), Heidelberg, Germany; 347 German Cancer Consortium (DKTK), German Cancer Research Center (DKFZ), Heidelberg, Germany; 348 National Center for Tumor Diseases (NCT) Heidelberg, Heidelberg, Germany; 349 Center for Biological Sequence Analysis, Department of Bio and Health Informatics, Technical University of Denmark, Lyngby, Denmark; 350 Novo Nordisk Foundation Center for Protein Research, University of Copenhagen, Copenhagen, Denmark; 351 Institute for Molecular Bioscience, University of Queensland, St. Lucia, Brisbane, QLD Australia; 352 Biomedical Engineering, Oregon Health and Science University, Portland, OR USA; 353 Division of Theoretical Bioinformatics, German Cancer Research Center (DKFZ), Heidelberg, Germany; 354 Institute of Pharmacy and Molecular Biotechnology and BioQuant, Heidelberg University, Heidelberg, Germany; 355 Federal Ministry of Education and Research, Berlin, Germany; 356 Melanoma Institute Australia, University of Sydney, Sydney, NSW Australia; 357 Pediatric Hematology and Oncology, University Hospital Muenster, Muenster, Germany; 358 Department of Pathology, Johns Hopkins University School of Medicine, Baltimore, MD USA; 359 McKusick-Nathans Institute of Genetic Medicine, Sidney Kimmel Comprehensive Cancer Center at Johns Hopkins University School of Medicine, Baltimore, MD USA; 360 Foundation Medicine, Inc, Cambridge, MA USA; 361 Department of Biomedical Data Science, Stanford University School of Medicine, Stanford, CA USA; 362 Department of Genetics, Stanford University School of Medicine, Stanford, CA USA; 363 Bakar Computational Health Sciences Institute and Department of Pediatrics, University of California, San Francisco, CA USA; 364 Institute of Clinical Medicine, Faculty of Medicine, University of Oslo, Oslo, Norway; 365 National Cancer Institute, National Institutes of Health, Bethesda, MD USA; 366 Royal Marsden NHS Foundation Trust, London and Sutton, UK; 367 Genome Biology Unit, European Molecular Biology Laboratory (EMBL), Heidelberg, Germany; 368 Department of Oncology, University of Cambridge, Cambridge, UK; 369 Li Ka Shing Centre, Cancer Research UK Cambridge Institute, University of Cambridge, Cambridge, UK; 370 Institut Gustave Roussy, Villejuif, France; 371 Cambridge University Hospitals NHS Foundation Trust, Cambridge, UK; 372 Department of Haematology, University of Cambridge, Cambridge, UK; 373 Anatomia Patológica, Hospital Clinic, Institut d’Investigacions Biomèdiques August Pi i Sunyer (IDIBAPS), University of Barcelona, Barcelona, Spain; 374 Spanish Ministry of Science and Innovation, Madrid, Spain; 375 University of Michigan Comprehensive Cancer Center, Ann Arbor, MI USA; 376 Department for BioMedical Research, University of Bern, Bern, Switzerland; 377 Department of Medical Oncology, Inselspital, University Hospital and University of Bern, Bern, Switzerland; 378 Graduate School for Cellular and Biomedical Sciences, University of Bern, Bern, Switzerland; 379 University of Pavia, Pavia, Italy; 380 University of Alabama at Birmingham, Birmingham, AL USA; 381 UHN Program in BioSpecimen Sciences, Toronto General Hospital, Toronto, ON Canada; 382 Department of Urology, Icahn School of Medicine at Mount Sinai, New York, NY USA; 383 Centre for Law and Genetics, University of Tasmania, Sandy Bay Campus, Hobart, TAS Australia; 384 Faculty of Biosciences, Heidelberg University, Heidelberg, Germany; 385 Department of Biochemistry, Microbiology and Immunology, Faculty of Medicine, University of Ottawa, Ottawa, ON Canada; 386 Division of Anatomic Pathology, Mayo Clinic, Rochester, MN USA; 387 Division of Cancer Epidemiology and Genetics, National Cancer Institute, National Institutes of Health, Bethesda, MD USA; 388 Illawarra Shoalhaven Local Health District L3 Illawarra Cancer Care Centre, Wollongong Hospital, Wollongong, NSW Australia; 389 BioForA, French National Institute for Agriculture, Food, and Environment (INRAE), ONF, Orléans, France; 390 Department of Biostatistics, Bloomberg School of Public Health, Johns Hopkins University, Baltimore, MD USA; 391 University of California San Diego, San Diego, CA USA; 392 Division of Experimental Pathology, Mayo Clinic, Rochester, MN USA; 393 Centre for Cancer Research, The Westmead Institute for Medical Research, University of Sydney, Sydney, NSW Australia; 394 Department of Gynaecological Oncology, Westmead Hospital, Sydney, NSW Australia; 395 PDXen Biosystems Inc, Seoul, South Korea; 396 Korea Advanced Institute of Science and Technology, Daejeon, South Korea; 397 Electronics and Telecommunications Research Institute, Daejeon, South Korea; 398 Institut National du Cancer (INCA), Boulogne-Billancourt, France; 399 Department of Genetics, Informatics Institute, University of Alabama at Birmingham, Birmingham, AL USA; 400 Division of Medical Oncology, National Cancer Centre, Singapore, Singapore; 401 Medical Oncology, University and Hospital Trust of Verona, Verona, Italy; 402 Department of Pediatrics, University Hospital Schleswig-Holstein, Kiel, Germany; 403 Hepatobiliary/Pancreatic Surgical Oncology Program, University Health Network, Toronto, ON Canada; 404 School of Biological Sciences, University of Auckland, Auckland, New Zealand; 405 Department of Surgery, University of Melbourne, Parkville, VIC Australia; 406 The Murdoch Children’s Research Institute, Royal Children’s Hospital, Parkville, VIC Australia; 407 Walter and Eliza Hall Institute, Parkville, VIC Australia; 408 Vancouver Prostate Centre, Vancouver, Canada; 409 Lunenfeld-Tanenbaum Research Institute, Mount Sinai Hospital, Toronto, ON Canada; 410 University of East Anglia, Norwich, UK; 411 Norfolk and Norwich University Hospital NHS Trust, Norwich, UK; 412 Victorian Institute of Forensic Medicine, Southbank, VIC Australia; 413 Department of Biomedical Informatics, Harvard Medical School, Boston, MA USA; 414 Department of Chemistry, Centre for Molecular Science Informatics, University of Cambridge, Cambridge, UK; 415 Ludwig Center at Harvard Medical School, Boston, MA USA; 416 Human Genome Sequencing Center, Baylor College of Medicine, Houston, TX USA; 417 Peter MacCallum Cancer Centre, University of Melbourne, Melbourne, VIC Australia; 418 Physics Division, Optimization and Systems Biology Lab, Massachusetts General Hospital, Boston, MA USA; 419 Department of Medicine, Baylor College of Medicine, Houston, TX USA; 420 University of Cologne, Cologne, Germany; 421 International Genomics Consortium, Phoenix, AZ USA; 422 Genomics Research Program, Ontario Institute for Cancer Research, Toronto, ON Canada; 423 Barking Havering and Redbridge University Hospitals NHS Trust, Romford, UK; 424 Children’s Hospital at Westmead, University of Sydney, Sydney, NSW Australia; 425 Department of Medicine, Section of Endocrinology, University and Hospital Trust of Verona, Verona, Italy; 426 Computational Biology Center, Memorial Sloan Kettering Cancer Center, New York, NY USA; 427 Department of Biology, ETH Zurich, Zürich, Switzerland; 428 Department of Computer Science, ETH Zurich, Zurich, Switzerland; 429 SIB Swiss Institute of Bioinformatics, Lausanne, Switzerland; 430 Weill Cornell Medical College, New York, NY USA; 431 Academic Department of Medical Genetics, University of Cambridge, Addenbrooke’s Hospital, Cambridge, UK; 432 MRC Cancer Unit, University of Cambridge, Cambridge, UK; 433 Departments of Pediatrics and Genetics, University of North Carolina at Chapel Hill, Chapel Hill, NC USA; 434 Seven Bridges Genomics, Charlestown, MA USA; 435 Annai Systems, Inc, Carlsbad, CA USA; 436 Department of Pathology, General Hospital of Treviso, Department of Medicine, University of Padua, Treviso, Italy; 437 Department of Computational Biology, University of Lausanne, Lausanne, Switzerland; 438 Department of Genetic Medicine and Development, University of Geneva Medical School, Geneva, CH Switzerland; 439 Swiss Institute of Bioinformatics, University of Geneva, Geneva, CH Switzerland; 440 The Francis Crick Institute, London, UK; 441 University of Leuven, Leuven, Belgium; 442 Institute of Medical Genetics and Applied Genomics, University of Tübingen, Tübingen, Germany; 443 Computational and Systems Biology, Genome Institute of Singapore, Singapore, Singapore; 444 School of Computing, National University of Singapore, Singapore, Singapore; 445 Big Data Institute, Li Ka Shing Centre, University of Oxford, Oxford, UK; 446 Biomedical Data Science Laboratory, Francis Crick Institute, London, UK; 447 Bioinformatics Group, Department of Computer Science, University College London, London, UK; 448 The Edward S. Rogers Sr. Department of Electrical and Computer Engineering, University of Toronto, Toronto, ON Canada; 449 Breast Cancer Translational Research Laboratory JC Heuson, Institut Jules Bordet, Brussels, Belgium; 450 Department of Oncology, Laboratory for Translational Breast Cancer Research, KU Leuven, Leuven, Belgium; 451 Institute for Research in Biomedicine (IRB Barcelona), The Barcelona Institute of Science and Technology, Barcelona, Spain; 452 Research Program on Biomedical Informatics, Universitat Pompeu Fabra, Barcelona, Spain; 453 Division of Medical Oncology, Princess Margaret Cancer Centre, Toronto, ON Canada; 454 Department of Physiology and Biophysics, Weill Cornell Medicine, New York, NY USA; 455 Institute for Computational Biomedicine, Weill Cornell Medicine, New York, NY USA; 456 Department of Pathology, UPMC Shadyside, Pittsburgh, PA USA; 457 Independent Consultant, Wellesley, USA; 458 Department of Cell and Molecular Biology, Science for Life Laboratory, Uppsala University, Uppsala, Sweden; 459 Department of Medicine and Department of Genetics, Washington University School of Medicine, St. Louis, St. Louis, MO USA; 460 Hefei University of Technology, Anhui, China; 461 Translational Cancer Research Unit, GZA Hospitals St.-Augustinus, Center for Oncological Research, Faculty of Medicine and Health Sciences, University of Antwerp, Antwerp, Belgium; 462 Simon Fraser University, Burnaby, BC Canada; 463 University of Pennsylvania, Philadelphia, PA USA; 464 Faculty of Science and Technology, University of Vic—Central University of Catalonia (UVic-UCC), Vic, Spain; 465 The Wellcome Trust, London, UK; 466 The Hospital for Sick Children, Toronto, ON Canada; 467 Department of Pathology, Queen Elizabeth University Hospital, Glasgow, UK; 468 Department of Genetics and Computational Biology, QIMR Berghofer Medical Research Institute, Brisbane, QLD Australia; 469 Department of Oncology, Centre for Cancer Genetic Epidemiology, University of Cambridge, Cambridge, UK; 470 Department of Public Health and Primary Care, Centre for Cancer Genetic Epidemiology, University of Cambridge, Cambridge, UK; 471 Prostate Cancer Canada, Toronto, ON Canada; 472 University of Cambridge, Cambridge, UK; 473 Department of Laboratory Medicine, Translational Cancer Research, Lund University Cancer Center at Medicon Village, Lund University, Lund, Sweden; 474 Heidelberg University, Heidelberg, Germany; 475 New BIH Digital Health Center, Berlin Institute of Health (BIH) and Charité - Universitätsmedizin Berlin, Berlin, Germany; 476 CIBER Epidemiología y Salud Pública (CIBERESP), Madrid, Spain; 477 Research Group on Statistics, Econometrics and Health (GRECS), UdG, Barcelona, Spain; 478 Quantitative Genomics Laboratories (qGenomics), Barcelona, Spain; 479 Icelandic Cancer Registry, Icelandic Cancer Society, Reykjavik, Iceland; 480 State Key Laboratory of Cancer Biology, and Xijing Hospital of Digestive Diseases, Fourth Military Medical University, Shaanxi, China; 481 Department of Medicine (DIMED), Surgical Pathology Unit, University of Padua, Padua, Italy; 482 Rigshospitalet, Copenhagen, Denmark; 483 Center for Cancer Genomics, National Cancer Institute, National Institutes of Health, Bethesda, MD USA; 484 Department of Biochemistry and Molecular Medicine, University of Montreal, Montreal, QC Canada; 485 Australian Institute of Tropical Health and Medicine, James Cook University, Douglas, QLD Australia; 486 Department of Neuro-Oncology, Istituto Neurologico Besta, Milano, Italy; 487 Bioplatforms Australia, North Ryde, NSW Australia; 488 Department of Pathology (Research), University College London Cancer Institute, London, UK; 489 Department of Surgical Oncology, Princess Margaret Cancer Centre, Toronto, ON Canada; 490 Department of Medical Oncology, Josephine Nefkens Institute and Cancer Genomics Centre, Erasmus Medical Center, Rotterdam, CN The Netherlands; 491 The University of Queensland Thoracic Research Centre, The Prince Charles Hospital, Brisbane, QLD Australia; 492 CIBIO/InBIO - Research Center in Biodiversity and Genetic Resources, Universidade do Porto, Vairão, Portugal; 493 HCA Laboratories, London, UK; 494 University of Liverpool, Liverpool, UK; 495 The Azrieli Faculty of Medicine, Bar-Ilan University, Safed, Israel; 496 Department of Neurosurgery, University of Florida, Gainesville, FL USA; 497 Department of Pathology, Graduate School of Medicine, University of Tokyo, Tokyo, Japan; 498 University of Milano Bicocca, Monza, Italy; 499 BGI-Shenzhen, Shenzhen, China; 500 Department of Pathology, Oslo University Hospital Ulleval, Oslo, Norway; 501 Center for Biomedical Informatics, Harvard Medical School, Boston, MA USA; 502 Department Biochemistry and Molecular Biomedicine, University of Barcelona, Barcelona, Spain; 503 Office of Cancer Genomics, National Cancer Institute, National Institutes of Health, Bethesda, MD USA; 504 Cancer Epigenomics, German Cancer Research Center (DKFZ), Heidelberg, Germany; 505 Department of Cancer Biology, The University of Texas MD Anderson Cancer Center, Houston, TX USA; 506 Department of Surgical Oncology, The University of Texas MD Anderson Cancer Center, Houston, TX USA; 507 Department of Computer Science, Yale University, New Haven, CT USA; 508 Department of Molecular Biophysics and Biochemistry, Yale University, New Haven, CT USA; 509 Program in Computational Biology and Bioinformatics, Yale University, New Haven, CT USA; 510 Center for Cancer Research, Massachusetts General Hospital, Boston, MA USA; 511 Department of Pathology, Massachusetts General Hospital, Boston, MA USA; 512 Department of Pathology, Memorial Sloan Kettering Cancer Center, New York, NY USA; 513 Division of Gastroenterology and Hepatology, Mayo Clinic, Rochester, MN USA; 514 University of Sydney, Sydney, NSW Australia; 515 University of Oxford, Oxford, UK; 516 Department of Surgery, Academic Urology Group, University of Cambridge, Cambridge, UK; 517 Department of Medicine II, University of Würzburg, Wuerzburg, Germany; 518 Sylvester Comprehensive Cancer Center, University of Miami, Miami, FL USA; 519 Institut Hospital del Mar d’Investigacions Mèdiques (IMIM), Barcelona, Spain; 520 Genome Integrity and Structural Biology Laboratory, National Institute of Environmental Health Sciences (NIEHS), Durham, NC USA; 521 St. Thomas’s Hospital, London, UK; 522 Osaka International Cancer Center, Osaka, Japan; 523 Department of Pathology, Skåne University Hospital, Lund University, Lund, Sweden; 524 Department of Medical Oncology, Beatson West of Scotland Cancer Centre, Glasgow, UK; 525 National Human Genome Research Institute, National Institutes of Health, Bethesda, MD USA; 526 Centre for Cancer Research, Victorian Comprehensive Cancer Centre, University of Melbourne, Melbourne, VIC Australia; 527 Department of Medicine, Section of Hematology/Oncology, University of Chicago, Chicago, IL USA; 528 German Center for Infection Research (DZIF), Partner Site Hamburg-Borstel-Lübeck-Riems, Hamburg, Germany; 529 Bioinformatics Research Centre (BiRC), Aarhus University, Aarhus, Denmark; 530 Department of Biotechnology, Ministry of Science and Technology, Government of India, New Delhi, Delhi India; 531 National Cancer Centre Singapore, Singapore, Singapore; 532 Brandeis University, Waltham, MA USA; 533 Department of Urologic Sciences, University of British Columbia, Vancouver, BC Canada; 534 Department of Internal Medicine, Stanford University, Stanford, CA USA; 535 The University of Texas Health Science Center at Houston, Houston, TX USA; 536 Imperial College NHS Trust, Imperial College, London, INY UK; 537 Senckenberg Institute of Pathology, University of Frankfurt Medical School, Frankfurt, Germany; 538 Department of Medicine, Division of Biomedical Informatics, UC San Diego School of Medicine, San Diego, CA USA; 539 Center for Precision Health, School of Biomedical Informatics, The University of Texas Health Science Center, Houston, TX USA; 540 Oxford Nanopore Technologies, New York, NY USA; 541 Institute of Medical Science, University of Tokyo, Tokyo, Japan; 542 Howard Hughes Medical Institute, University of California Santa Cruz, Santa Cruz, CA USA; 543 Wakayama Medical University, Wakayama, Japan; 544 Department of Internal Medicine, Division of Medical Oncology, Lineberger Comprehensive Cancer Center, University of North Carolina at Chapel Hill, Chapel Hill, NC USA; 545 University of Tennessee Health Science Center for Cancer Research, Memphis, TN USA; 546 Department of Histopathology, Salford Royal NHS Foundation Trust, Salford, UK; 547 Faculty of Biology, Medicine and Health, University of Manchester, Manchester, UK; 548 BIOPIC, ICG and College of Life Sciences, Peking University, Beijing, China; 549 Peking-Tsinghua Center for Life Sciences, Peking University, Beijing, China; 550 Children’s Hospital of Philadelphia, Philadelphia, PA USA; 551 Department of Bioinformatics and Computational Biology and Department of Systems Biology, The University of Texas MD Anderson Cancer Center, Houston, TX USA; 552 Karolinska Institute, Stockholm, Sweden; 553 The Donnelly Centre, University of Toronto, Toronto, ON Canada; 554 Department of Medical Genetics, College of Medicine, Hallym University, Chuncheon, South Korea; 555 Department of Experimental and Health Sciences, Institute of Evolutionary Biology (UPF-CSIC), Universitat Pompeu Fabra, Barcelona, Spain; 556 Health Data Science Unit, University Clinics, Heidelberg, Germany; 557 Massachusetts General Hospital Center for Cancer Research, Charlestown, MA USA; 558 Hokkaido University, Sapporo, Japan; 559 Department of Pathology and Clinical Laboratory, National Cancer Center Hospital, Tokyo, Japan; 560 Department of Genetics, University of North Carolina at Chapel Hill, Chapel Hill, NC USA; 561 Computational Biology, Leibniz Institute on Aging - Fritz Lipmann Institute (FLI), Jena, Germany; 562 University of Melbourne Centre for Cancer Research, Melbourne, VIC Australia; 563 University of Nebraska Medical Center, Omaha, NE USA; 564 Syntekabio Inc, Daejeon, South Korea; 565 Department of Pathology, Academic Medical Center, Amsterdam, AZ The Netherlands; 566 China National GeneBank-Shenzhen, Shenzhen, China; 567 Division of Molecular Genetics, German Cancer Research Center (DKFZ), Heidelberg, Germany; 568 Division of Life Science and Applied Genomics Center, Hong Kong University of Science and Technology, Clear Water Bay, Hong Kong, China; 569 Icahn School of Medicine at Mount Sinai, New York, NY USA; 570 Geneplus-Shenzhen, Shenzhen, China; 571 School of Computer Science and Technology, Xi’an Jiaotong University, Xi’an, China; 572 AbbVie, North Chicago, IL USA; 573 Institute of Pathology, Charité – University Medicine Berlin, Berlin, Germany; 574 Centre for Translational and Applied Genomics, British Columbia Cancer Agency, Vancouver, BC Canada; 575 Edinburgh Royal Infirmary, Edinburgh, UK; 576 Berlin Institute for Medical Systems Biology, Max Delbrück Center for Molecular Medicine, Berlin, Germany; 577 Department of Pediatric Immunology, Hematology and Oncology, University Hospital, Heidelberg, Germany; 578 German Cancer Research Center (DKFZ), Heidelberg, Germany; 579 Heidelberg Institute for Stem Cell Technology and Experimental Medicine (HI-STEM), Heidelberg, Germany; 580 Institute for Computational Biomedicine, Weill Cornell Medical College, New York, NY USA; 581 New York Genome Center, New York, NY USA; 582 Department of Urology, James Buchanan Brady Urological Institute, Johns Hopkins University School of Medicine, Baltimore, MD USA; 583 Department of Preventive Medicine, Graduate School of Medicine, The University of Tokyo, Tokyo, Japan; 584 Department of Molecular and Cellular Biology, Baylor College of Medicine, Houston, TX USA; 585 Department of Pathology and Immunology, Baylor College of Medicine, Houston, TX USA; 586 Michael E. DeBakey Veterans Affairs Medical Center, Houston, TX USA; 587 Technical University of Denmark, Lyngby, Denmark; 588 Department of Pathology, College of Medicine, Hanyang University, Seoul, South Korea; 589 Academic Unit of Surgery, School of Medicine, College of Medical, Veterinary and Life Sciences, University of Glasgow, Glasgow Royal Infirmary, Glasgow, UK; 590 Department of Pathology, Asan Medical Center, College of Medicine, Ulsan University, Songpa-gu, Seoul South Korea; 591 Science Writer, Garrett Park, MD USA; 592 International Cancer Genome Consortium (ICGC)/ICGC Accelerating Research in Genomic Oncology (ARGO) Secretariat, Ontario Institute for Cancer Research, Toronto, ON Canada; 593 University of Ljubljana, Ljubljana, Slovenia; 594 Department of Public Health Sciences, University of Chicago, Chicago, IL USA; 595 Research Institute, NorthShore University HealthSystem, Evanston, IL USA; 596 Department for Biomedical Research, University of Bern, Bern, Switzerland; 597 Centre of Genomics and Policy, McGill University and Génome Québec Innovation Centre, Montreal, QC Canada; 598 Carolina Center for Genome Sciences, University of North Carolina at Chapel Hill, Chapel Hill, NC USA; 599 Hopp Children’s Cancer Center (KiTZ), Heidelberg, Germany; 600 Pediatric Glioma Research Group, German Cancer Research Center (DKFZ), Heidelberg, Germany; 601 Cancer Research UK, London, UK; 602 Indivumed GmbH, Hamburg, Germany; 603 Genome Integration Data Center, Syntekabio, Inc, Daejeon, South Korea; 604 University Hospital Zurich, Zurich, Switzerland; 605 Clinical Bioinformatics, Swiss Institute of Bioinformatics, Geneva, Switzerland; 606 Institute for Pathology and Molecular Pathology, University Hospital Zurich, Zurich, Switzerland; 607 Institute of Molecular Life Sciences, University of Zurich, Zurich, Switzerland; 608 MRC Human Genetics Unit, MRC IGMM, University of Edinburgh, Edinburgh, UK; 609 Women’s Cancer Program at the Samuel Oschin Comprehensive Cancer Institute, Cedars-Sinai Medical Center, Los Angeles, CA USA; 610 Department of Biology, Bioinformatics Group, Division of Molecular Biology, Faculty of Science, University of Zagreb, Zagreb, Croatia; 611 Department for Internal Medicine II, University Hospital Schleswig-Holstein, Kiel, Germany; 612 Genetics and Molecular Pathology, SA Pathology, Adelaide, SA Australia; 613 Department of Gastric Surgery, National Cancer Center Hospital, Tokyo, Japan; 614 Department of Bioinformatics, Division of Cancer Genomics, National Cancer Center Research Institute, Tokyo, Japan; 615 A.A. Kharkevich Institute of Information Transmission Problems, Moscow, Russia; 616 Oncology and Immunology, Dmitry Rogachev National Research Center of Pediatric Hematology, Moscow, Russia; 617 Skolkovo Institute of Science and Technology, Moscow, Russia; 618 Department of Surgery, The George Washington University, School of Medicine and Health Science, Washington, DC USA; 619 Endocrine Oncology Branch, Center for Cancer Research, National Cancer Institute, National Institutes of Health, Bethesda, MD USA; 620 Melanoma Institute Australia, Macquarie University, Sydney, NSW Australia; 621 MIT Computer Science and Artificial Intelligence Laboratory, Massachusetts Institute of Technology, Cambridge, MA USA; 622 Tissue Pathology and Diagnostic Oncology, Royal Prince Alfred Hospital, Sydney, NSW Australia; 623 Cholangiocarcinoma Screening and Care Program and Liver Fluke and Cholangiocarcinoma Research Centre, Faculty of Medicine, Khon Kaen University, Khon Kaen, Thailand; 624 Controlled Department and Institution, New York, NY USA; 625 Englander Institute for Precision Medicine, Weill Cornell Medicine, New York, NY USA; 626 National Cancer Center, Gyeonggi, South Korea; 627 Department of Biochemistry, College of Medicine, Ewha Womans University, Seoul, South Korea; 628 Health Sciences Department of Biomedical Informatics, University of California San Diego, La Jolla, CA USA; 629 Research Core Center, National Cancer Centre Korea, Goyang-si, South Korea; 630 Department of Health Sciences and Technology, Sungkyunkwan University School of Medicine, Seoul, South Korea; 631 Samsung Genome Institute, Seoul, South Korea; 632 Breast Oncology Program, Dana-Farber/Brigham and Women’s Cancer Center, Boston, MA USA; 633 Department of Surgery, Memorial Sloan Kettering Cancer Center, New York, NY USA; 634 Division of Breast Surgery, Brigham and Women’s Hospital, Boston, MA USA; 635 Integrative Bioinformatics Support Group, National Institute of Environmental Health Sciences (NIEHS), Durham, NC USA; 636 Department of Clinical Science, University of Bergen, Bergen, Norway; 637 Center For Medical Innovation, Seoul National University Hospital, Seoul, South Korea; 638 Department of Internal Medicine, Seoul National University Hospital, Seoul, South Korea; 639 Institute of Computer Science, Polish Academy of Sciences, Warsawa, Poland; 640 Functional and Structural Genomics, German Cancer Research Center (DKFZ), Heidelberg, Germany; 641 Laboratory of Translational Genomics, Division of Cancer Epidemiology and Genetics, National Cancer Institute, , National Institutes of Health, Bethesda, MD USA; 642 Institute for Medical Informatics Statistics and Epidemiology, University of Leipzig, Leipzig, Germany; 643 Morgan Welch Inflammatory Breast Cancer Research Program and Clinic, The University of Texas MD Anderson Cancer Center, Houston, TX USA; 644 Department of Hematology and Oncology, Georg-Augusts-University of Göttingen, Göttingen, Germany; 645 Institute of Cell Biology (Cancer Research), University of Duisburg-Essen, Essen, Germany; 646 King’s College London and Guy’s and St. Thomas’ NHS Foundation Trust, London, UK; 647 Center for Epigenetics, Van Andel Research Institute, Grand Rapids, MI USA; 648 The University of Queensland Centre for Clinical Research, Royal Brisbane and Women’s Hospital, Herston, QLD Australia; 649 Department of Pediatric Oncology and Hematology, University of Cologne, Cologne, Germany; 650 University of Düsseldorf, Düsseldorf, Germany; 651 Department of Pathology, Institut Jules Bordet, Brussels, Belgium; 652 Institute of Biomedicine, Sahlgrenska Academy at University of Gothenburg, Gothenburg, Sweden; 653 Children’s Medical Research Institute, Sydney, NSW Australia; 654 ILSbio, LLC Biobank, Chestertown, MD USA; 655 Division of Genetics and Genomics, Boston Children’s Hospital, Harvard Medical School, Boston, MA USA; 656 Institute for Bioengineering and Biopharmaceutical Research (IBBR), Hanyang University, Seoul, South Korea; 657 Department of Statistics, University of California Santa Cruz, Santa Cruz, CA USA; 658 National Genotyping Center, Institute of Biomedical Sciences, Academia Sinica, Taipei, Taiwan; 659 Department of Vertebrate Genomics/Otto Warburg Laboratory Gene Regulation and Systems Biology of Cancer, Max Planck Institute for Molecular Genetics, Berlin, Germany; 660 McGill University and Genome Quebec Innovation Centre, Montreal, QC Canada; 661 biobyte solutions GmbH, Heidelberg, Germany; 662 Gynecologic Oncology, NYU Laura and Isaac Perlmutter Cancer Center, New York University, New York, NY USA; 663 Division of Oncology, Stem Cell Biology Section, Washington University School of Medicine, St. Louis, MO USA; 664 Department of Systems Biology, The University of Texas MD Anderson Cancer Center, Houston, TX USA; 665 Harvard University, Cambridge, MA USA; 666 Urologic Oncology Branch, Center for Cancer Research, National Cancer Institute, National Institutes of Health, Bethesda, MD USA; 667 University of Oslo, Oslo, Norway; 668 University of Toronto, Toronto, ON Canada; 669 Peking University, Beijing, China; 670 School of Life Sciences, Peking University, Beijing, China; 671 Leidos Biomedical Research, Inc, McLean, VA USA; 672 Hematology, Hospital Clinic, Institut d’Investigacions Biomèdiques August Pi i Sunyer (IDIBAPS), University of Barcelona, Barcelona, Spain; 673 Second Military Medical University, Shanghai, China; 674 Chinese Cancer Genome Consortium, Shenzhen, China; 675 Department of Medical Oncology, Beijing Hospital, Beijing, China; 676 Laboratory of Molecular Oncology, Key Laboratory of Carcinogenesis and Translational Research (Ministry of Education), Peking University Cancer Hospital and Institute, Beijing, China; 677 School of Medicine/School of Mathematics and Statistics, University of St. Andrews, St, Andrews, Fife UK; 678 Institute for Systems Biology, Seattle, WA USA; 679 Department of Biochemistry and Molecular Biology, Faculty of Medicine, University Institute of Oncology-IUOPA, Oviedo, Spain; 680 Institut Bergonié, Bordeaux, France; 681 Cancer Unit, MRC University of Cambridge, Cambridge, UK; 682 Department of Pathology and Laboratory Medicine, Center for Personalized Medicine, Children’s Hospital Los Angeles, Los Angeles, CA USA; 683 John Curtin School of Medical Research, Canberra, ACT Australia; 684 MVZ Department of Oncology, PraxisClinic am Johannisplatz, Leipzig, Germany; 685 Department of Information Technology, Ghent University, Ghent, Belgium; 686 Department of Plant Biotechnology and Bioinformatics, Ghent University, Ghent, Belgium; 687 Institute for Genomic Medicine, Nationwide Children’s Hospital, Columbus, OH USA; 688 Computational Biology Program, School of Medicine, Oregon Health and Science University, Portland, OR USA; 689 Department of Surgery, Duke University, Durham, NC USA; 690 Institució Catalana de Recerca i Estudis Avançats (ICREA), Barcelona, Spain; 691 Institut Català de Paleontologia Miquel Crusafont, Universitat Autònoma de Barcelona, Barcelona, Spain; 692 University of Glasgow, Glasgow, UK; 693 Institut d’Investigacions Biomèdiques August Pi i Sunyer (IDIBAPS), Barcelona, Spain; 694 Division of Oncology, Washington University School of Medicine, St. Louis, MO USA; 695 Department of Surgery and Cancer, Imperial College, London, INY UK; 696 Applications Department, Oxford Nanopore Technologies, Oxford, UK; 697 Department of Obstetrics, Gynecology and Reproductive Services, University of California San Francisco, San Francisco, CA USA; 698 Department of Biochemistry and Molecular Medicine, University California at Davis, Sacramento, CA USA; 699 STTARR Innovation Facility, Princess Margaret Cancer Centre, Toronto, ON Canada; 700 Discipline of Surgery, Western Sydney University, Penrith, NSW Australia; 701 Yale School of Medicine, Yale University, New Haven, CT USA; 702 Department of Genetics, Lineberger Comprehensive Cancer Center, University of North Carolina at Chapel Hill, Chapel Hill, NC USA; 703 Departments of Neurology and Neurosurgery, Henry Ford Hospital, Detroit, MI USA; 704 Precision Oncology, OHSU Knight Cancer Institute, Oregon Health and Science University, Portland, OR USA; 705 Institute of Pathology, University Medical Center Hamburg-Eppendorf, Hamburg, Germany; 706 Department of Health Sciences, Faculty of Medical Sciences, Kyushu University, Fukuoka, Japan; 707 Heidelberg Academy of Sciences and Humanities, Heidelberg, Germany; 708 Department of Clinical Pathology, University of Melbourne, Melbourne, VIC, Australia; 709 Department of Pathology, Roswell Park Cancer Institute, Buffalo, NY USA; 710 Department of Computer Science, University of Helsinki, Helsinki, Finland; 711 Institute of Biotechnology, University of Helsinki, Helsinki, Finland; 712 Organismal and Evolutionary Biology Research Programme, University of Helsinki, Helsinki, Finland; 713 Department of Obstetrics and Gynecology, Division of Gynecologic Oncology, Washington University School of Medicine, St. Louis, MO USA; 714 Penrose St. Francis Health Services, Colorado Springs, CO USA; 715 Institute of Pathology, Ulm University and University Hospital of Ulm, Ulm, Germany; 716 National Cancer Center, Tokyo, Japan; 717 Genome Institute of Singapore, Singapore, Singapore; 718 32Program in Computational Biology and Bioinformatics, Yale University, New Haven, CT USA; 719 German Cancer Aid, Bonn, Germany; 720 Programme in Cancer and Stem Cell Biology, Centre for Computational Biology, Duke-NUS Medical School, Singapore, Singapore; 721 The Chinese University of Hong Kong, Shatin, NT, Hong Kong China; 722 Fourth Military Medical University, Shaanxi, China; 723 The University of Cambridge School of Clinical Medicine, Cambridge, UK; 724 St. Jude Children’s Research Hospital, Memphis, TN USA; 725 University Health Network, Princess Margaret Cancer Centre, Toronto, ON Canada; 726 Center for Biomolecular Science and Engineering, University of California Santa Cruz, Santa Cruz, CA USA; 727 Department of Medicine, University of Chicago, Chicago, IL USA; 728 Department of Neurology, Mayo Clinic, Rochester, MN USA; 729 Cambridge Oesophagogastric Centre, Cambridge University Hospitals NHS Foundation Trust, Cambridge, UK; 730 Department of Computer Science, Carleton College, Northfield, MN USA; 731 Institute of Cancer Sciences, College of Medical Veterinary and Life Sciences, University of Glasgow, Glasgow, UK; 732 Department of Epidemiology, University of Alabama at Birmingham, Birmingham, AL USA; 733 HudsonAlpha Institute for Biotechnology, Huntsville, AL USA; 734 O’Neal Comprehensive Cancer Center, University of Alabama at Birmingham, Birmingham, AL USA; 735 Department of Pathology, Keio University School of Medicine, Tokyo, Japan; 736 Department of Hepatobiliary and Pancreatic Oncology, National Cancer Center Hospital, Tokyo, Japan; 737 Sage Bionetworks, Seattle, WA USA; 738 Lymphoma Genomic Translational Research Laboratory, National Cancer Centre, Singapore, Singapore; 739 Department of Clinical Pathology, Robert-Bosch-Hospital, Stuttgart, Germany; 740 Department of Cell and Systems Biology, University of Toronto, Toronto, ON Canada; 741 Department of Biosciences and Nutrition, Karolinska Institutet, Stockholm, Sweden; 742 Center for Liver Cancer, Research Institute and Hospital, National Cancer Center, Gyeonggi, South Korea; 743 Division of Hematology-Oncology, Samsung Medical Center, Sungkyunkwan University School of Medicine, Seoul, South Korea; 744 Samsung Advanced Institute for Health Sciences and Technology, Sungkyunkwan University School of Medicine, Seoul, South Korea; 745 Cheonan Industry-Academic Collaboration Foundation, Sangmyung University, Cheonan, South Korea; 746 NYU Langone Medical Center, New York, NY USA; 747 Department of Hematology and Medical Oncology, Cleveland Clinic, Cleveland, OH USA; 748 Department of Radiation Oncology, University of California San Francisco, San Francisco, CA USA; 749 Department of Health Sciences Research, Mayo Clinic, Rochester, MN USA; 750 Helen F. Graham Cancer Center at Christiana Care Health Systems, Newark, DE USA; 751 Heidelberg University Hospital, Heidelberg, Germany; 752 CSRA Incorporated, Fairfax, VA USA; 753 Research Department of Pathology, University College London Cancer Institute, London, UK; 754 Department of Research Oncology, Guy’s Hospital, King’s Health Partners AHSC, King’s College London School of Medicine, London, UK; 755 Faculty of Medicine and Health Sciences, Macquarie University, Sydney, NSW Australia; 756 University Hospital of Minjoz, INSERM UMR 1098, Besançon, France; 757 Spanish National Cancer Research Centre, Madrid, Spain; 758 Center of Digestive Diseases and Liver Transplantation, Fundeni Clinical Institute, Bucharest, Romania; 759 Cureline, Inc, South San Francisco, CA USA; 760 St. Luke’s Cancer Centre, Royal Surrey County Hospital NHS Foundation Trust, Guildford, UK; 761 Cambridge Breast Unit, Addenbrooke’s Hospital, Cambridge University Hospital NHS Foundation Trust and NIHR Cambridge Biomedical Research Centre, Cambridge, UK; 762 East of Scotland Breast Service, Ninewells Hospital, Aberdeen, UK; 763 Department of Genetics, Microbiology and Statistics, University of Barcelona, IRSJD, IBUB, Barcelona, Spain; 764 Department of Obstetrics and Gynecology, Medical College of Wisconsin, Milwaukee, WI USA; 765 Hematology and Medical Oncology, Winship Cancer Institute of Emory University, Atlanta, GA USA; 766 Department of Computer Science, Princeton University, Princeton, NJ USA; 767 Vanderbilt Ingram Cancer Center, Vanderbilt University, Nashville, TN USA; 768 Ohio State University College of Medicine and Arthur G. James Comprehensive Cancer Center, Columbus, OH USA; 769 Department of Surgery, Yokohama City University Graduate School of Medicine, Kanagawa, Japan; 770 Division of Chromatin Networks, German Cancer Research Center (DKFZ) and BioQuant, Heidelberg, Germany; 771 Research Computing Center, University of North Carolina at Chapel Hill, Chapel Hill, NC USA; 772 School of Molecular Biosciences and Center for Reproductive Biology, Washington State University, Pullman, WA USA; 773 Finsen Laboratory and Biotech Research and Innovation Centre (BRIC), University of Copenhagen, Copenhagen, Denmark; 774 Department of Laboratory Medicine and Pathobiology, University of Toronto, Toronto, ON Canada; 775 Department of Pathology, Human Oncology and Pathogenesis Program, Memorial Sloan Kettering Cancer Center, New York, NY USA; 776 University Hospital Giessen, Pediatric Hematology and Oncology, Giessen, Germany; 777 Oncologie Sénologie, ICM Institut Régional du Cancer, Montpellier, France; 778 Institute of Clinical Molecular Biology, Christian-Albrechts-University, Kiel, Germany; 779 Institute of Pathology, University of Wuerzburg, Wuerzburg, Germany; 780 Department of Urology, North Bristol NHS Trust, Bristol, UK; 781 SingHealth, Duke-NUS Institute of Precision Medicine, National Heart Centre Singapore, Singapore, Singapore; 782 Department of Computer Science, University of Toronto, Toronto, ON Canada; 783 Bern Center for Precision Medicine, University Hospital of Bern, University of Bern, Bern, Switzerland; 784 Englander Institute for Precision Medicine, Weill Cornell Medicine and New York Presbyterian Hospital, New York, NY USA; 785 Meyer Cancer Center, Weill Cornell Medicine, New York, NY USA; 786 Pathology and Laboratory, Weill Cornell Medical College, New York, NY USA; 787 Vall d’Hebron Institute of Oncology: VHIO, Barcelona, Spain; 788 General and Hepatobiliary-Biliary Surgery, Pancreas Institute, University and Hospital Trust of Verona, Verona, Italy; 789 National Centre for Biological Sciences, Tata Institute of Fundamental Research, Bangalore, India; 790 Indiana University, Bloomington, IN USA; 791 Department of Pathology, GZA-ZNA Hospitals, Antwerp, Belgium; 792 Analytical Biological Services, Inc, Wilmington, DE USA; 793 Sydney Medical School, University of Sydney, Sydney, NSW Australia; 794 cBio Center, Dana-Farber Cancer Institute, Harvard Medical School, Boston, MA USA; 795 Department of Cell Biology, Harvard Medical School, Boston, MA USA; 796 Advanced Centre for Treatment Research and Education in Cancer, Tata Memorial Centre, Navi Mumbai, Maharashtra India; 797 School of Environmental and Life Sciences, Faculty of Science, The University of Newcastle, Ourimbah, NSW Australia; 798 Department of Dermatology, University Hospital of Essen, Essen, Germany; 799 Bioinformatics and Omics Data Analytics, German Cancer Research Center (DKFZ), Heidelberg, Germany; 800 Department of Urology, Charité Universitätsmedizin Berlin, Berlin, Germany; 801 Martini-Clinic, Prostate Cancer Center, University Medical Center Hamburg-Eppendorf, Hamburg, Germany; 802 Department of General Internal Medicine, University of Kiel, Kiel, Germany; 803 German Cancer Consortium (DKTK), Partner site Berlin, Berlin, Germany; 804 Cancer Research Institute, Beth Israel Deaconess Medical Center, Boston, MA USA; 805 University of Pittsburgh, Pittsburgh, PA USA; 806 Department of Ophthalmology and Ocular Genomics Institute, Massachusetts Eye and Ear, Harvard Medical School, Boston, MA USA; 807 Center for Psychiatric Genetics, NorthShore University HealthSystem, Evanston, IL USA; 808 Van Andel Research Institute, Grand Rapids, MI USA; 809 Laboratory of Molecular Medicine, Human Genome Center, Institute of Medical Science, University of Tokyo, Tokyo, Japan; 810 Japan Agency for Medical Research and Development, Tokyo, Japan; 811 Korea University, Seoul, South Korea; 812 Murtha Cancer Center, Walter Reed National Military Medical Center, Bethesda, MD USA; 813 Human Genetics, University of Kiel, Kiel, Germany; 814 Department of Oncologic Pathology, Dana-Farber Cancer Institute, Harvard Medical School, Boston, MA USA; 815 Oregon Health and Science University, Portland, OR USA; 816 Center for RNA Interference and Noncoding RNA, The University of Texas MD Anderson Cancer Center, Houston, TX USA; 817 Department of Experimental Therapeutics, The University of Texas MD Anderson Cancer Center, Houston, TX USA; 818 Department of Gynecologic Oncology and Reproductive Medicine, The University of Texas MD Anderson Cancer Center, Houston, TX USA; 819 University Hospitals Coventry and Warwickshire NHS Trust, Coventry, UK; 820 Department of Radiation Oncology, Radboud University Nijmegen Medical Centre, Nijmegen, GA The Netherlands; 821 Institute for Genomics and Systems Biology, University of Chicago, Chicago, IL USA; 822 Clinic for Hematology and Oncology, St.-Antonius-Hospital, Eschweiler, Germany; 823 Computational and Systems Biology Program, Memorial Sloan Kettering Cancer Center, New York, NY USA; 824 University of Iceland, Reykjavik, Iceland; 825 Division of Computational Genomics and Systems Genetics, German Cancer Research Center (DKFZ), Heidelberg, Germany; 826 Dundee Cancer Centre, Ninewells Hospital, Dundee, UK; 827 Department for Internal Medicine III, University of Ulm and University Hospital of Ulm, Ulm, Germany; 828 Institut Curie, INSERM Unit 830, Paris, France; 829 Department of Gastroenterology and Hepatology, Yokohama City University Graduate School of Medicine, Kanagawa, Japan; 830 Department of Laboratory Medicine, Radboud University Nijmegen Medical Centre, Nijmegen, GA The Netherlands; 831 Division of Cancer Genome Research, German Cancer Research Center (DKFZ), Heidelberg, Germany; 832 Department of General Surgery, Singapore General Hospital, Singapore, Singapore; 833 Cancer Science Institute of Singapore, National University of Singapore, Singapore, Singapore; 834 Department of Medical and Clinical Genetics, Genome-Scale Biology Research Program, University of Helsinki, Helsinki, Finland; 835 East Anglian Medical Genetics Service, Cambridge University Hospitals NHS Foundation Trust, Cambridge, UK; 836 Irving Institute for Cancer Dynamics, Columbia University, New York, NY USA; 837 Institute of Molecular and Cell Biology, Singapore, Singapore; 838 Laboratory of Cancer Epigenome, Division of Medical Science, National Cancer Centre Singapore, Singapore, Singapore; 839 Universite Lyon, INCa-Synergie, Centre Léon Bérard, Lyon, France; 840 Department of Urology, Mayo Clinic, Rochester, MN USA; 841 Royal National Orthopaedic Hospital - Stanmore, Stanmore, Middlesex UK; 842 Department of Biochemistry, Genetics and Immunology, University of Vigo, Vigo, Spain; 843 Giovanni Paolo II / I.R.C.C.S. Cancer Institute, Bari, BA Italy; 844 Neuroblastoma Genomics, German Cancer Research Center (DKFZ), Heidelberg, Germany; 845 Fondazione Policlinico Universitario Gemelli IRCCS, Rome, Italy, Rome, Italy; 846 University of Verona, Verona, Italy; 847 Centre National de Génotypage, CEA - Institute de Génomique, Evry, France; 848 CAPHRI Research School, Maastricht University, Maastricht, ER The Netherlands; 849 Department of Biopathology, Centre Léon Bérard, Lyon, France; 850 Université Claude Bernard Lyon 1, Villeurbanne, France; 851 Core Research for Evolutional Science and Technology (CREST), JST, Tokyo, Japan; 852 Department of Biological Sciences, Laboratory for Medical Science Mathematics, Graduate School of Science, University of Tokyo, Yokohama, Japan; 853 Department of Medical Science Mathematics, Medical Research Institute, Tokyo Medical and Dental University (TMDU), Tokyo, Japan; 854 Cancer Ageing and Somatic Mutation Programme, Wellcome Sanger Institute, Hinxton, UK; 855 University Hospitals Birmingham NHS Foundation Trust, Birmingham, UK; 856 Centre for Cancer Research and Cell Biology, Queen’s University, Belfast, UK; 857 Breast Medical Oncology, The University of Texas MD Anderson Cancer Center, Houston, TX USA; 858 Department of Surgery, Johns Hopkins University School of Medicine, Baltimore, MD USA; 859 Department of Oncology-Pathology, Science for Life Laboratory, Karolinska Institute, Stockholm, Sweden; 860 School of Cancer Sciences, Faculty of Medicine, University of Southampton, Southampton, UK; 861 Department of Gene Technology, Tallinn University of Technology, Tallinn, Estonia; 862 Genetics and Genome Biology Program, SickKids Research Institute, The Hospital for Sick Children, Toronto, ON Canada; 863 Departments of Neurosurgery and Hematology and Medical Oncology, Winship Cancer Institute and School of Medicine, Emory University, Atlanta, GA USA; 864 Department of Clinical and Molecular Medicine, Faculty of Medicine and Health Sciences, Norwegian University of Science and Technology, Trondheim, Norway; 865 Argmix Consulting, North Vancouver, BC Canada; 866 Department of Information Technology, Ghent University, Interuniversitair Micro-Electronica Centrum (IMEC), Ghent, Belgium; 867 Nuffield Department of Surgical Sciences, John Radcliffe Hospital, University of Oxford, Oxford, UK; 868 Institute of Mathematics and Computer Science, University of Latvia, Riga, LV Latvia; 869 Discipline of Pathology, Sydney Medical School, University of Sydney, Sydney, NSW Australia; 870 Department of Applied Mathematics and Theoretical Physics, Centre for Mathematical Sciences, University of Cambridge, Cambridge, UK; 871 Department of Epidemiology and Biostatistics, Memorial Sloan Kettering Cancer Center, New York, NY USA; 872 Department of Statistics, Columbia University, New York, NY USA; 873 Department of Immunology, Genetics and Pathology, Science for Life Laboratory, Uppsala University, Uppsala, Sweden; 874 School of Electronic and Information Engineering, Xi’an Jiaotong University, Xi’an, China; 875 Department of Histopathology, Cambridge University Hospitals NHS Foundation Trust, Cambridge, UK; 876 Oxford NIHR Biomedical Research Centre, University of Oxford, Oxford, UK; 877 Georgia Regents University Cancer Center, Augusta, GA USA; 878 Wythenshawe Hospital, Manchester, UK; 879 Department of Genetics, Washington University School of Medicine, St.Louis, MO USA; 880 Department of Biological Oceanography, Leibniz Institute of Baltic Sea Research, Rostock, Germany; 881 Wellcome Centre for Human Genetics, University of Oxford, Oxford, UK; 882 Department of Molecular and Human Genetics, Baylor College of Medicine, Houston, TX USA; 883 Thoracic Oncology Laboratory, Mayo Clinic, Rochester, MN USA; 884 Institute for Genomic Medicine, Nationwide Children’s Hospital, Columbus, OH USA; 885 Department of Obstetrics and Gynecology, Division of Gynecologic Oncology, Mayo Clinic, Rochester, MN USA; 886 International Institute for Molecular Oncology, Poznań, Poland; 887 Poznan University of Medical Sciences, Poznań, Poland; 888 Genomics and Proteomics Core Facility High Throughput Sequencing Unit, German Cancer Research Center (DKFZ), Heidelberg, Germany; 889 NCCS-VARI Translational Research Laboratory, National Cancer Centre Singapore, Singapore, Singapore; 890 Edison Family Center for Genome Sciences and Systems Biology, Washington University, St. Louis, MO USA; 891 MRC-University of Glasgow Centre for Virus Research, Glasgow, UK; 892 Department of Medical Informatics and Clinical Epidemiology, Division of Bioinformatics and Computational Biology, OHSU Knight Cancer Institute, Oregon Health and Science University, Portland, OR USA; 893 School of Electronic Information and Communications, Huazhong University of Science and Technology, Wuhan, China; 894 Department of Applied Mathematics and Statistics, Johns Hopkins University, Baltimore, MD USA; 895 Department of Cancer Genome Informatics, Graduate School of Medicine, Osaka University, Osaka, Japan; 896 Institute of Computer Science, Heidelberg University, Heidelberg, Germany; 897 School of Mathematics and Statistics, University of Sydney, Sydney, NSW Australia; 898 Ben May Department for Cancer Research, University of Chicago, Chicago, IL USA; 899 Department of Human Genetics, University of Chicago, Chicago, IL USA; 900 Tri-Institutional PhD Program in Computational Biology and Medicine, Weill Cornell Medicine, New York, NY USA; 901 The First Affiliated Hospital, Xi’an Jiaotong University, Xi’an, China; 902 Department of Medicine and Therapeutics, The Chinese University of Hong Kong, Shatin, NT, Hong Kong China; 903 Department of Biostatistics, The University of Texas MD Anderson Cancer Center, Houston, TX USA; 904 Duke-NUS Medical School, Singapore, Singapore; 905 Department of Surgery, Ruijin Hospital, Shanghai Jiaotong University School of Medicine, Shanghai, China; 906 School of Computing Science, University of Glasgow, Glasgow, UK; 907 Division of Orthopaedic Surgery, Oslo University Hospital, Oslo, Norway; 908 Eastern Clinical School, Monash University, Melbourne, VIC Australia; 909 Epworth HealthCare, Richmond, VIC Australia; 910 Department of Biostatistics and Computational Biology, Dana-Farber Cancer Institute and Harvard Medical School, Boston, MA USA; 911 Department of Biomedical Informatics, College of Medicine, The Ohio State University, Columbus, OH USA; 912 The Ohio State University Comprehensive Cancer Center (OSUCCC – James), Columbus, OH USA; 913 The University of Texas School of Biomedical Informatics (SBMI) at Houston, Houston, TX USA; 914 Department of Biostatistics, University of North Carolina at Chapel Hill, Chapel Hill, NC USA; 915 Department of Biochemistry and Molecular Genetics, Feinberg School of Medicine, Northwestern University, Chicago, IL USA; 916 Faculty of Medicine and Health, University of Sydney, Sydney, NSW Australia; 917 Department of Pathology, Erasmus Medical Center Rotterdam, Rotterdam, GD The Netherlands; 918 Division of Molecular Carcinogenesis, The Netherlands Cancer Institute, Amsterdam, CX The Netherlands; 919 Institute of Molecular Life Sciences and Swiss Institute of Bioinformatics, University of Zurich, Zurich, Switzerland

**Keywords:** Cancer genetics, Computational models

## Abstract

The type and genomic context of cancer mutations depend on their causes. These causes have been characterized using signatures that represent mutation types that co-occur in the same tumours. However, it remains unclear how mutation processes change during cancer evolution due to the lack of reliable methods to reconstruct evolutionary trajectories of mutational signature activity. Here, as part of the ICGC/TCGA Pan-Cancer Analysis of Whole Genomes (PCAWG) Consortium, which aggregated whole-genome sequencing data from 2658 cancers across 38 tumour types, we present TrackSig, a new method that reconstructs these trajectories using optimal, joint segmentation and deconvolution of mutation type and allele frequencies from a single tumour sample. In simulations, we find TrackSig has a 3–5% activity reconstruction error, and 12% false detection rate. It outperforms an aggressive baseline in situations with branching evolution, CNA gain, and neutral mutations. Applied to data from 2658 tumours and 38 cancer types, TrackSig permits pan-cancer insight into evolutionary changes in mutational processes.

## Abstract (web summary)

Cancers evolve as they progress under differing selective pressures. Here, as part of the ICGC/TCGA Pan-Cancer Analysis of Whole Genomes (PCAWG) Consortium, the authors present the method TrackSig the estimates evolutionary trajectories of somatic mutational processes from single bulk tumour data.

## Introduction

Somatic mutations accumulate throughout our lifetime, arising from external sources or from processes intrinsic to the cell^1,2^. Some sources generate characteristic patterns of mutations. For example, smoking is associated with G–T mutations; UV radiation is associated with C to T mutations^3–5^. Some processes provide a constant source of mutations^6^ while others are sporadic^7^.

One can estimate the contribution of different mutation processes to the collection of somatic mutations present in a sample through mutational signature analysis. In this type of analysis, single nucleotide variants (SNVs) are classified into 96 types based on the type of substitution and tri-nucleotide context (e.g. ACG–ATG)^2^. Mutational signatures across the 96 types were derived by non-negative matrix factorization in the previous work by Alexandrov et al.^2^ . Many of the signatures are associated with known mutational processes including smoking^7^, non-homologous double strand break repair^2^, and ionizing radiation^8^. The activities of some signatures are correlated with patient age^6^ and suggest their use as a molecular clock^9^. Thus, signature analysis can identify the DNA damage repair pathways that are absent in cancer, can predict prognosis^10^, or guide treatment choice^11^.

Formally, a “mutational signature” is a probability distribution over a categorical variable representing a mutation type, where each element is a probability of generating a mutation from the corresponding type^12^. Each signature is assigned an “activity” (also called “exposure”) which represents the proportion of mutations that the signature generates. Activities for pre-defined signatures can be computed from the total mutational spectrum of a sample by using constrained regression^13,14^.

Mutational sources can change over time^15–19^. Mutations caused by carcinogen activity stop accumulating when the activity ends^7^. Mutations associated with defective DNA damage repair, such as BRCA1 loss^1,2^ will begin to accumulate after that loss. Recent analyses of sequencing data from single bulk samples have reported modest changes in signature activities between clonal and subclonal populations^9,20^ based on groups of mutations identified by clustering their variant allele frequencies (VAFs). However, the accuracy of these methods relies heavily on the sensitivity and precision of this clustering, which is typically low^21,22^ except, in some cases, in multi-region sequencing studies^15–19,23^.

Here we introduce TrackSig, a new method to reconstruct signature activities across time without VAF clustering. We use VAF to approximately order mutations based on their prevalence within the cancer cell population and then track changes in signature activity that are consistent with this ordering.

We use realistic simulations and bootstrap analysis to help assess the accuracy of signature activity reconstructions under a variety of different evolutionary scenarios. Using TrackSig and Pan-cancer Analysis of Whole Genomes (PCAWG) dataset of 2658 cancers, we have previously demonstrated^24^ that signature activities change often during the lifetime of a cancer. Here we show that these changes can often be a more sensitive indicator of new subclonal lineages than VAF clustering.

The PCAWG Consortium aggregated whole-genome sequencing data from 2658 cancers across 38 tumour types generated by the ICGC and TCGA projects. These sequencing data were re-analysed with standardised, high-accuracy pipelines to align to the human genome (reference build hs37d5) and identify germline variants and somatically acquired mutations, as described by PCAWG Network^25^.

## Results

In this paper we perform the realistic simulations to evaluate TrackSig’s performance at reconstructing signature activities, detecting the number of mutation clusters, and correctly placing the changepoints under different scenarios including violations of TrackSig’s assumptions.

**Fig. 1:**
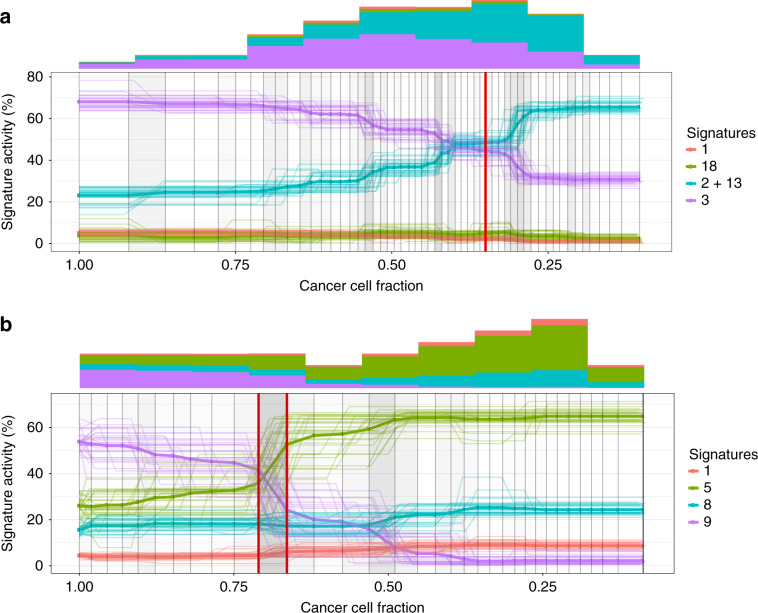
Signature activity trajectories for two samples. Each plot is constructed from VAF data from a single tumour sample. Each line is an activity trajectory that depicts inferred activities for a single signature (*y*-axis) as a function of decreasing CCF (*x*-axis). The thin lines are trajectories from each of 30 bootstrap runs. The bold line depicts the mean activities across bootstraps. The vertical lines indicate time points in the original dataset, and are placed at the average CCF of their 100 associated mutations. Changes in activity trajectories are not necessarily aligned with vertical bars because mean CCFs of time points change across bootstraps. Frequency of changepoints between two vertical bars is indicated by shade, the darker shades indicate higher density of changepoints. Subclonal boundaries found by PCAWG consensus clustering^24^ are shown in red vertical lines. These boundaries are not used in trajectory calculation and are only shown for comparison. Histograms show the mutation counts per signature in fixed width intervals of CCF. **a** Breast cancer sample. In clonal signatures remains constant with dominating signature 3 (associated with BRCA1 mutations). In the subclone activity to signature 3 decreases and is replaced by SNVs associated with APOBEC/AID (signatures 2 and 13). **b** Chronic lymphocytic leukaemia sample. Signature 9 (somatic hypermutation) dominates during clonal expansion and drops from 55% activity to almost zero in the subclone. Signature 5 compensates for this change.

TrackSig was applied to the 2552 whole-genome sequencing samples with more than 600 SNVs contained within the white and grey lists of the PCAWG group. Here we provide methodological details of TrackSig’s use on real data (PCAWG). The analysis of signature trends and relation of changepoints found by TrackSig to subclonal boundaries is described elsewhere^24^. Figure 1 shows examples of TrackSig trajectories for two tumour samples (breast cancer and leukaemia).

### Choice of mutation signatures

By default, following Alexandrov et al.^2^, we classify mutations into 96 types based on their three-nucleotide context. Point mutations fall into six different mutation types (i.e. C → [AGT] and T → [ACG]) excluding complementary pairs. There are 16 (4 × 4) possible combinations of the 5′ and 3′ nucleotides. Thus, SNVs are separated into 96 (*K* = 16 × 6 = 96) types. However, TrackSig can use different mutation type labelling schemes, so long as the signatures and the mutation types are provided as input.

Within the context of PCAWG, we use the set of 48 single-base signatures (SBS) developed by PCAWG-Signature group. The first 30 of those signatures are slightly modified versions of original signatures defined by Alexandrov et al.^2,12^ and have the same numbering and interpretation. The original 30 signatures are described at COSMIC (http://cancer.sanger.ac.uk/cosmic/signatures). Signature analysis methods, including TrackSig, fit activities for only a subset of the signatures. These signatures are called the "active" signatures. The activities for the non-active signatures are clamped to zero. For example, SBS 7 has been detected almost exclusively in skin cancers and likely describes mutations caused by UV light^2^. As such, it is only assigned active status in skin cancers. In our analysis, we use the active signatures reported by PCAWG-Signature group. For analyses based on COSMIC signatures, one can use active signatures per cancer type as provided on COSMIC website. TrackSig can also be used to automatically select active signatures, as described in a later section.

### Simulations

We tested sensitivity of TrackSig in multiple error scenarios using simulated data with known ground truth. First, because signatures overlap in the mutation types that they can produce, we first test reconstruction accuracy when SNVs are accurately assigned to the time points to assess errors due to inability to correctly assign signature activity. We describe these non-parametric simulations in the next section.

In “Results” section, we assess reconstructions when the mutation ordering is inferred based on mutation VAF. In this scenario, reconstruction errors can occur when (i) cancer cell fraction (CCF) estimates are inaccurate and, (ii) there are two SNV clusters which overlap in CCF space but have different signature activity profiles. In the latter case, SNVs from both clusters will be located in the same or adjacent time point bins and will have a mixture of signature activity profiles from two clusters. To test reconstruction errors in these two scenarios, we produce clonal evolution simulations where we sample the VAF data from a clonal evolution model with binomial sequencing noise. To simulate the VAF detection limit for mutations imposed by somatic mutation calling, we remove any mutation with fewer than three variant reads.

Finally, as part of clonal evolution simulations, we assess model misspecification error by introducing violations of the assumptions of infinite sites and the relationship between CCF and timing of mutation occurrence. Also, in some simulations, we introduce mutations under neutral selection (i.e. neutrally evolving mutations). We computed the number and VAFs of these mutations using the model and effective mutation rates derived by Williams et al.^26,27^ as detailed in Supplementary Note 3. As a baseline, we compare TrackSig’s reconstruction error to the widely used strategy of first assigning SNVs to clusters based on VAF, then computing mutation signatures activities from the assigned mutations within each cluster.

### Non-parametric simulations

In the non-parametric simulations, we test the ideal scenario when SNVs are correctly ordered and assigned to the time point bins. Here we want to access the ability of TrackSig to reconstruct signature activities from the distribution of mutation types and place changepoints at the correct locations.

Each simulation has 50 time points, each time point is a bin of 100 mutations. This corresponds to the average number of somatic mutations detected in PCAWG. Each sample also contains four active signatures. Two of those signatures are 1 and 5, which are nearly always active in the PCAWG samples. For the remaining two signatures, we test all 1035 possible combinations of the other 46 signatures.

We generate simulations with 0–3 changepoints that are placed randomly on the timeline. For each segment on the timeline, we sample signature activities from a uniform distribution over activity vectors. Finally, we sample 100 mutation types per time point from the discrete distribution derived using the sampled activities as mixing coefficients for the four signatures.

Next, we run TrackSig on the simulated data and compare the reconstructed activity trajectories to the ground truth. We remove changepoints with small change, that is, where activities of all signatures change by <5% in reconstructed trajectories. This threshold is derived in “Results” section from permutation analysis.

**Fig. 2:**
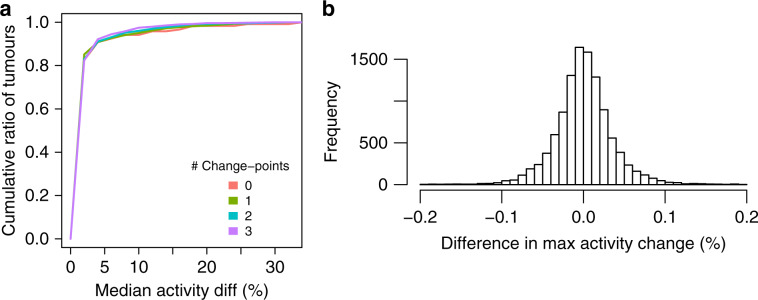
Results on non-parametric simulations. **a** Median activity difference between the reconstructed trajectories and the ground truth. Lines correspond to the simulations with 0, 1, 2, or 3 changepoints. The median in computed across all signatures and time points in the sample. **b** Distribution of maximum activity change (MAC) discrepancies between between estimated activities and ground truth.

We computed the absolute difference between predicted activities and the ground truth at each time point and take the median across all time points and all four signatures. We called this the median activity difference per simulation. On the simulations with no changepoints, the median of these median per simulation differences is 0.7%. On simulations with 1–3 changepoints, this median increases slightly to 2%. The cumulative distribution of the median per simulation differences is shown in Fig. 2.

For the PCAWG data, we report the maximum activity change (MAC) across activity trajectory^24^ The maximum change is the difference between maximum and minimum activity across all time points in a sample. We also report the direction of change (down if maximum occurs before minimum and up otherwise). Here, we evaluate TrackSig’s accuracy in these estimates on the simulated data. The MAC discrepancies between the estimated and ground-truth trajectories is <5% in 83.2% of cases across all signatures in all simulations (Fig. 2b).

To compare the direction of the activity change, we divide signatures into those with: decreasing activity, increasing activity, and no activity change (i.e. max absolute change is <5%). The direction of maximum change is consistent in 95.2% of all signatures across all simulations.

To compute the number of false positives and false negatives, we count a true positive detection if at least one of predicted changepoints occur within three time points of an actual one. A false negative is when no predicted changepoints are within three time points of an actual change. This criteria is identical to the one we use to evaluate whether a changepoint supports a subclonal boundary^24^. We deem a predicted changepoint a false positive if it occurs more than three time points away from the closest actual changepoint.

Tables 1 and 2 show the percentage of simulations where we observe a certain number of false negatives, and false positives respectively (see Supplementary Table 1 for additional results). On average, there are 0.12 false positives per simulation and 0.02 false negatives on average per simulation.

**Table 1:** False negatives rates in non-parametric simulations.

No. of true changepoints		0	1	2	3
Avg no. of FN per simulation		0.0	0.008	0.038	0.058
No. of false negative changepoints (FN)	0	1	0.992	0.962	0.947
	1	0	0.008	0.038	0.049
	2	0	0	0	0.003

Each cell shows the proportion of simulations that have certain number of false negatives (normalized within the column). See main text for definition of positive and negative time points. The first row of the table shows the average number of false negatives per simulation

**Table 2:** False positives rates in non-parametric simulations.

No. of true changepoints		0	1	2	3
Avg No. of FP per simulation		0.130	0.128	0.118	0.116
No. of false positive changepoints (FP)	0	0.909	0.896	0.9	0.889
	1	0.06	0.083	0.087	0.106
	2	0.024	0.019	0.011	0.005
	3	0.005	0.002	0.002	0
	4	0.002	0	0.001	0

Each cell shows the proportion of simulations that have certain number of false positives (normalized within the column). See main text for definition of positive and negative time points. The first row of the table shows the average number of false positives per simulation

### Clonal evolution simulations

Generating realistic simulated data requires making some assumptions about how tumours evolve. In this section, we simulate VAF data consistent with clonal evolution theory^28^, with some small violations. Specifically, every mutation belongs to one of a small set of subclones. If the CCFs of each mutation could be estimated precisely, then mutation CCF data consistent with clonal evolution theory would have signature activities that are piece-wise constant functions of CCF. These VAF data are thus consistent with all previous work estimating signature activities.

We also include some simulations that violate the clonal evolution assumptions and test TrackSig’s robustness to these violations. We performed six different simulations, described briefly here (see Supplementary Note 3 for details). We generated 100 simulations of each type.

First, we aim to evaluate false negative and false positive rates of identifying subclones via TrackSig. We simulated VAF data from (a) one clonal population and no subclones (b) one clonal population and one subclone with a variety of CCF values sampled from a uniform distribution, assuming a linear clonal tree. We sample the variant allele counts for the mutations in accordance to the cluster CCFs from a binomial distribution. We create simulations with four signatures—age-related signatures 1 and 5 and two other randomly-chosen signatures, which we will refer to as A1 and A2. The activities of A1 and A2 are sampled uniformly in each clone, under the constraint that at least one of them has a signature change of at least 30%. We sample mutation types from the signature mixture treating it as a multinomial distribution. We simulated mean read depths of 10, 30, and 100.

The performance of TrackSig was also assessed under conditions of neutral evolution. We sampled mutation VAFs as per the previous paragraph but we also added some neutrally evolving mutations to the clonal cluster. We determined the number of neutral mutations to add and sampled their VAFs according to the model from the Williams et al.^26^ (see Supplementary Note 3). The signature activities for the neutral mutations were the same as those of the other mutations associated with the clonal cluster.

To assess TrackSig’s accuracy when the timeline does not reflect the ordering of acquistion of SNVs, we generated VAF data from a branched phylogeny. In branching simulation we generated VAF data assuming a branching clonal tree with two subclones. We force the sum of subclonal CCFs to be <1, otherwise the infinite sites assumption will be violated^1^. A later occurring subclone with a different signature activity profile has a higher CCF than a subclone with a profile matching the clonal fraction. We sample mutation VAFs and mutation types for the clusters similarly to the one- and two-cluster cases.

We next assess TrackSig’s accuracy for reconstructing activity changes when SNV VAFs are affected by a copy number aberration (CNA). We generated VAF data with a clonal CNA gain affecting 10% of the SNV VAFs. In 5% of the mutations the CNA gain is affecting the mutant allele and in 5% the CNA gain is affecting the reference allele. This simulation is created similarly to the branching with three clusters. The difference is that we modify the probability of sampling a mutant allele to consider the altered mutant and reference copy numbers.

Finally, we create simulations with violation of infinite site assumption, where the same mutations independently occurred in two branched subclones. To model this, we set the CCFs of 3% of mutations to be equal to the sum of CCFs from the two subclones.

We compared results of TrackSig to the widely used approach of first clustering mutations by CCF and then inferring signature activities within each cluster^15–19^. We perform the clustering using SciClone^29^. We do a hard assignment of mutations to clusters detected by SciClone, and use DeconstructSigs^13^ to estimate signature activities within these clusters. We report the results with two clustering methods in SciClone: Beta mixture model (BMM, default) and Beta-binomial mixture model (Binomial BMM). Note that the beta-binomial is an exact match to the noise model used in our data simulation, so we expect excellent performance from SciClone. We use the Beta model to simulate inaccuracies due to incorrect noise model specification.

### Simulation results

We compared TrackSig and Sciclone + DeconstructSigs pipeline (hereafter SciClone for brevity, see Supplementary Note 4) against the ground truth in the simulation across the seven simulation types and depths 10, 30, and 100. First, we computed the median activity error over all mutations in the five types of non-neutral simulations, see Supplementary Fig. 7. In Supplementary Fig. 8 we compared the errors in the neutral one- and two-cluster cases. Across all depths, the majority (83.6%) of TrackSig reconstructions have <0.05 median error with ground truth versus 55.0% in SciClone. On average TrackSig’s activity error is 4.5%; SciClone’s is 6.9% across all seven simulations. Measuring activity error using KL divergence gives similar results (Supplementary Fig. 4), and TrackSig’s accuracy is relatively insensitive to bin size at multiple depths (see Supplementary Figs. 5 and 6)

**Fig. 3:**
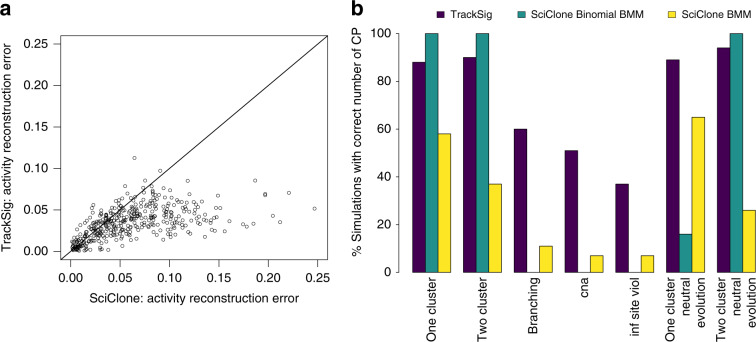
TrackSig and SciClone performance on clonal evolution simulations. **a** Scatterplot of median activity errors (i.e. absolute activity difference) on all depth 30 simulations (see Supplementary Fig. 3 for depths 10 and 100). Mean activity error: TrackSig 3.5%, SciClone 6.2%. **b** Grouped barplot shows proportion of simulations where each method predicts the correct number of subclones for different simulation types as indicated on *x*-axis label. Different SciClone bars indicate different noise model selections. Results are for the simulations of average depth 30. Results for depths 10 and 100 are shown in Supplementary Fig. 2.

We then compared methods based on their ability to detect subclones. Figure 3b shows the percentage of simulations when each method predicted correct number of subclones for depth 30 (see Supplementary Fig. 2 for depths 10 and 100). For this comparison, we used two different noise models with SciClone: the binomial-beta model which is an exact match to how the simulated data are generated, and the beta model, which is not and which was the model used for computing the reconstruction error above.

As expected, the SciClone binomial-beta performs nearly perfectly on the depth 30 simulations which match the assumptions of this model (Fig 3b, one-cluster, two-clusters). However, the binomial-beta model is fragile, and performs poorly when its assumptions are violated (Fig. 3b, branching, cna, inf sites viol, one-cluster neutral evolution). The beta model does not perform well under the ideal situation, but it is better than the binomial model when the clonal evolution assumptions are violated. In contrast, TrackSig retains the ~10% false positive rate in calling subclones observed in the non-parametric simulations. TrackSig’s high performance is maintained also in the neutral mutation simulations. In the other violation scenarios TrackSig has much better performance than either of the two SciClone noise models.

**Fig. 4:**
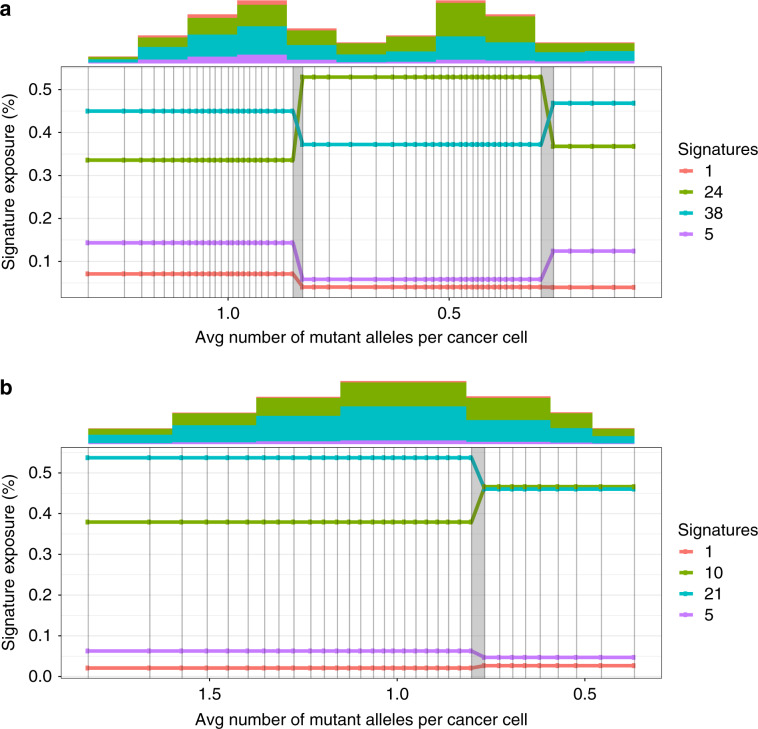
TrackSig reconstruction examples. **a** Simulated data was generated with two clusters and clonal neutral mutations at read depth 100. TrackSig incorrectly places a changepoint before a cluster of neutral mutations from the clonal lineage near the VAF detection limit. However, because the signature activities match those in the clonal cluster, this error could be detected and corrected in post-processing. **b** Simulated data was generated with two clusters at read depth 10. TrackSig correctly identifies one changepoint. Although the simulation contains two clusters, there is only a single mode of CCF, thus making CCF-cluster-based detection of subclones impossible. However, the histogram on top shows that there are differences in mutation type distributions between the left and right tails, permitting TrackSig to correctly identify a changepoint. Both figures use an expanded *x*-axis that shows the whole spread of estimated CCF, this is indicated with a change in the *x*-label descriptor.

Supplementary Fig. 2 shows that at depth 100, TrackSig has ~90% accuracy in all scenarios except the two-cluster neutral evolution simulation. This scenario is particularly difficult because the clonal cluster is split with about 500 neutral mutations from the clonal lineage clustered at the VAF detection limit; so the mutation type distributions actually have two clear changepoints: one going from the clonal lineage to the subclonal one, and then another returning to the clonal (Fig. 4a). It may be possible to detect this error in post-processing (see “Discussion” Section). Note, however, that this simulation may not be representative of real data because, unlike other simulations^27^, we are not simulating neutral mutations from the subclone. The depth 10 simulations are particularly challenging as well, because the VAF distributions of the clonal and subclonal clusters overlap substantially, making it difficult to detect multiple clusters. Here we see that TrackSig is an even more sensitive detector of a second cluster than the correct SciClone binomial model, see Fig. 4b. None of the methods do well in the branching, CNA, and infinite site violation scenarios because they require the detection of three clusters. Performance in the neutral evolution scenarios here matches that in the non-neutral ones because there are very few neutral mutations above the VAF detection limit. In summary, TrackSig is more robust to violations of the clonal evolution assumptions that are made by most subclonal reconstruction algorithms (see^23,27^).

### Methodology on real data

**Fig. 5:**
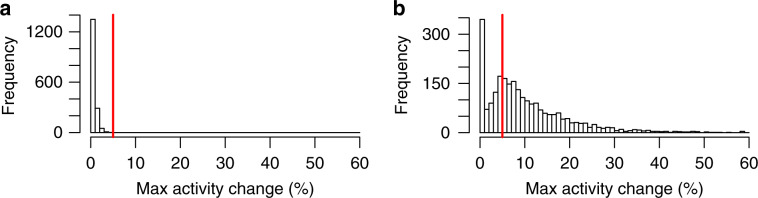
Maximum signature activity changes in PCAWG samples. The red line shows the threshold of 5%, above which we consider changes to be significant. **a** Changes on random orders of mutations where we do not expect to see change in activities. **b** activity changes in TrackSig trajectories across all samples (on mutations sorted by CCF). Frequency axis shows the number of samples where we observe the certain activity change.

We analyze the variation of signature activities on PCAWG data across time and across samples. We compute the maximum change of the signatures in each sample, which is simply the difference between maximum and minimum activity of the signature. To assess whether a signature change is statistically significant, we permute the mutations in each sample and run the trajectory estimation on the permuted set. Since permuted mutations are not sorted in time, we expect no change in the activity trajectories over time. The MAC that we observe on permuted set of mutations does not exceed 5% in any sample. Therefore, we only consider signature changes above 5% to be significant (Fig. 5).

### Bootstrapping

We assess the variability in activity trajectories by performing bootstrap on the PCAWG data. We sample mutations with replacement from the original set and re-calculate their activities and changepoints. We perform 30 bootstrap runs for each sample. Figure 1 shows examples of bootstrapped trajectories from two samples (breast cancer and leukaemia).

Signature trajectories calculated on bootstrap data are stable. The mean standard deviation of activity values calculated at each time point is 2.9%. We also evaluate the consistency of signature changes across the entire activity trajectory: size of signature change and location of the changepoint. The mean standard deviation of the change in signature activity is 5.3% across the bootstraps. This standard deviation does not exceed 5% in 55.8% of samples (does not exceed 10% in 94.3% of samples, Supplementary Fig. 1).

In TrackSig the number of changepoints calculated during activity fitting does vary across bootstrap samples. We observe 1.02 standard deviation in the number of changepoints. To assess the variability in the location of the changepoints, we matched nearby changepoints between bootstrap samples and measured their average distance in CCF. Because the number of changepoints can change between samples, as a reference, we randomly choose one of the samples that has a number of changepoints equal to the median number of changepoints among all samples. Then, in all other bootstrap runs, we match each changepoint to the closest run in the reference. We found that location of the changepoints is consistent across bootstraps: on average, changepoints are located 0.093 CCF apart from the closest reference changepoint.

### Signatures with most changing activities

**Fig. 6:**
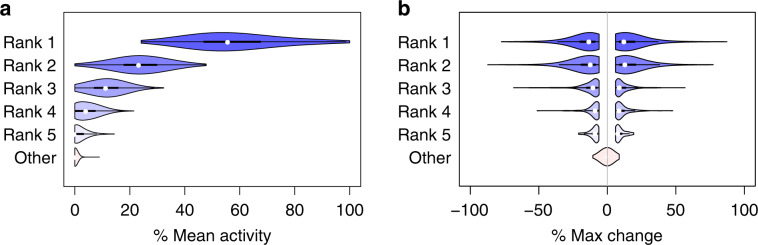
PCAWG signature changes by activity level. **a** Mean signature activities ranked from the largest to the smallest within each sample in PCAWG data. Only the top five signatures with the highest activities in a sample are shown. **b** Maximum changes of signature activities for the corresponding signatures on plot (**a**). The changes below 5% are omitted.

As shown by Fig. 6, samples typically have only two or three signatures with high activities. These signatures are usually the most variable (up to 87.2% max change, 12% on average). Other signature have low-activity and remain constant. On average 3.6% of overall activity is made up of low-activity signatures (with activity <5%). Low-activity signatures most likely appear due to the uncertainty of our signature activity estimates. The mean standard deviation of signature activities is 2.9%, thus, we remove signatures with activity <5% as they are within two standard deviations of 0%.

### Trends in signature change per cancer type

**Fig. 7:**
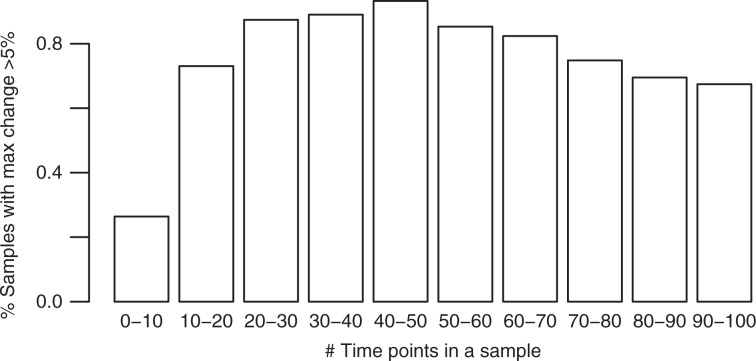
Frequency of activity change by number of mutations. Proportion of tumours that have a significant change greater than 5% activity depending on the number of time points in a sample. Each bar corresponds to the range of number of time points in a sample; each time point contains 100 mutations.

The majority of PCAWG samples have a signature change: 76.1% of samples have a max change >5% in at least one signature; 48.4% of samples have change >10%. However, the number of signature changes depends on the number of mutations in the sample. Out of samples with >10 time points only 26.3% of samples have a change >5% compared with 80.4% across the rest of the samples (see distribution on Fig. 7).

## Discussion

TrackSig reconstructs the evolutionary trajectories of mutational signature activities by sorting point mutations according to their inferred CCF and then partitioning this sorted list into groups of mutations with constant signature activities. TrackSig estimates uncertainty in the location of the changepoints using bootstrap. TrackSig is designed to be applied to VAF data on SNVs from a single sample, however, it can be applied to either sorted lists of point mutations derived from subclonal reconstruction algorithms, or CCFs from a single cancer sample derived from methods which perform multi-sample reconstructions or subclonal CNA reconstructions.

Changepoints often correspond to boundaries between subclones^24^. In our simulations we show that TrackSig often better detects subclones than methods explicitly designed to find subclones, especially when there is a mismatch between the assumed and actual VAF generation process. By reconstructing changes in signature activities, TrackSig can potentially help identify DNA damage repair processes disrupted in the cell and, in doing so, help inform treatment^11^.

Previous approaches estimate signature activities for a group of mutations without considering their timing (e.g. eMu^30^ or deconstructSigs^13^). Therefore, the attempts to compare activity changes across evolutionary history have relied on pre-defined groups of mutations, such as those occurring before or after whole-genome duplications^7,9,31,32^; those classified as clonal or subclonal^1,9^; or those grouped in subclones via multi-region sequencing^15–19^. As such, the accuracy of these methods relies on (i) the accuracy in grouping mutations based on VAF—which is low with data from a single bulk sample^21^; and (ii) the existence of a small number of subclones or mutation groups within a sample, which is not true for neutrally evolving tumours^23,26,33^.

In contrast, TrackSig uses the distributions of mutation types to group mutations, this permits more accurate reconstruction of signature activities than clustering mutations by VAF alone. Indeed, as our simulations demonstrate, not only are the signature activities more accurately reconstructed, but in some cases, TrackSig is a more sensitive detector of subclones. Furthermore, TrackSig makes fewer assumptions about the underlying VAF distribution, so it can be readily applied to data from neutrally evolving tumour populations^26,33^. Our simulations further demonstrate that TrackSig’s reconstructions are less sensitive to model misspecification errors, such as violations of the infinite sites assumptions.

Clustering methods applied to VAFs from single bulk samples require high read depth for accuracy^21^. Indeed, due to this challenge, previous approaches have used multi-region sequencing^15–19,23,33–36^. In contrast, TrackSig can be deployed in a much larger range of settings. Separately, we report that TrackSig can detect subclones that are missed by VAF clustering methods^24^

Another important innovation of TrackSig is the use of CCF as a surrogate for evolutionary timing. Similar ideas have been used in human population genetics, where variant allele frequency to get relative order of mutations along the ancestral lineage^37^. In population genetics, allele frequency is calculated across individuals, while we calculate VAF across cell population within a single sample. In TrackSig we estimate CCF and reconstructions of clonal CNAs. In “Methods” section, we discuss the validity of using CCF as a surrogate for evolutionary time.

In TrackSig, the number of mutation types is provided as a parameter and is not fixed to 96 types. Because of this, it is straightforward to generalize TrackSig to reconstruct the activities of different mutation signatures or different mutations, so long as these mutations can be approximately ordered by their evolutionary time and each mutation can be classified into one of a fixed number of categories. In this paper, we ordered SNVs by decreasing CCF. This same strategy could be naturally extended to indels for which the infinite sites assumption is also valid. The infinite sites assumption should also be valid for structural variants (SVs) associated with well-defined breakpoints, thus permitting TrackSig to be used to track activities to recently defined SV signatures^32^. The CCFs of SVs can be estimated using the VAFs of split-reads mapping to their breakpoints^38^. Because they cover larger genomic regions, infinite sites is less valid for CNAs, although it is possible to approximately order clonal CNAs based on the inferred multiplicity of SNVs affected by them^9^.

TrackSig also requires a pre-defined set of mutation signatures, each of which is a probability distribution over the mutation types. However, if these signatures are unavailable, they can be defined by non-negative matrix factorization, or Latent Dirichlet Allocation^39^, if counts across mutation types are available from multiple cancer samples.

TrackSig can be applied to VAFs from bulk sequencing data from multi-region sequencing or longitudinal samples by simply running it on each sample separately. In preliminary experiments testing this approach we found broad consistency in the active signature selected, and in the signature activities of the clonal mutations in each sample. We observe with only 0.03% mean absolute activity difference (0.017 KL divergence) between signature activities of clonal cluster across different samples. See Supplementary Note 5 and Supplementary Fig. 9 for details.

For ease of presentation, we have assumed that ordering SNVs by CCF recovers the order in which they accumulated in the genomes of ancestral cells. However, this assumption is not critical for correct reconstruction of signature activity changes.

First, we have shown through bootstrap sampling and the clonal evolution simulations that errors in the estimation of SNV CCFs due to sampling noise have a limited impact on TrackSig’s ability to estimate accurate activity trajectories. We have similarly shown that these activity trajectories are not impacted if a small fraction (3%) of the SNVs violate the infinite sites assumption.

However, these trajectories can be impacted by incorrect ordering of a large numbers of SNVs. These can occur in two ways. First, misordering can occur if a CNA changes the number of SNV allele’s per cell. For example, daughter cells can fail to inherit SNVs in their mother cells due to a loss of heterozygosity (LOH). If a CNA reconstruction is available, TrackSig will correct for any detected clonal LOH when ordering SNVs, and will not attempt to order SNVs in regions affected by subclonal CNAs, thereby resolving this difficulty. However, if a CNA reconstruction is not available, or it is inaccurate, the accuracy of the activity trajectories can suffer. As such, we recommend only using TrackSig when CNA reconstructions are available and reliable.

Second, SNV ordering need not correspond to the time of acquisition when a single sample contains SNVs from subclones from different branches of the cancer phylogeny. In these circumstances, there is not a single linear order for the activities, and furthermore, late occurring subclones on a different branch can have higher CCF than earlier ones occurring in the sample. This situation also occurs when the sample contains a large number of neutrally evolving mutations from multiple subclonal lineages, as seen in the two cluster, depth 100 simulations. Note that such circumstances are rare in single biopsies^31^ and that furthermore, a subclone can only be misordered if its CCF is <50% due to the Pigeonhole Principle^1^, so the ordering by CCFs in guaranteed to be correct up until 50% CCF. However, even when these misorderings occurs, our simulations demonstrate that, with one exception, TrackSig’s activity reconstructions, and estimation of number of subclones, are largely unaffected. Note, however, that the true complexity of tumour evolution with multiple subclones and high depth sequencing may confound the analysis in a way that is not assessed here, and so care must be taken interpreting results in tumours with complex clonal architectures.

Even in the rare circumstance that SNV misordering does occur, it may be possible to detect it, and interpret the activity changes correctly. For example, if late occurring but misordered SNVs manifest a more drastic change in signature activity, this misordering may be detectable by the presence of oscillations in the activity trajectories. To address this issue, when assessing overall change in signature activity, we computed the difference between the lowest and highest activities for each signature. This difference will be consistent regardless of ordering.

The timelines reconstructed by TrackSig are computed with a fixed number of mutations in each bin. If overall rate of generating mutations in tumour was constant, our timeline would correspond to the real time. However, tumour mutation rate often accelerates throughout development^40,41^. Although the changing rate does not affect our analysis, the estimates of the pseudo-time might not be linearly related to real time.

Estimating changes in overall mutation rate is difficult. A possible way to correct for this is to adjust the timeline based on activities of signatures 1 and 5. Some report that signatures 1 and 5 operate as a cellular clock as the number of mutations contributed by these signatures is proportional to the age of the individual^6^. Determining the association between our pseudo-time estimates and real time is left for further investigation.

Our method TrackSig provides further insight how signature profile changes throughout tumour development. We show that through signatures analysis we can detect major events in tumour evolution, notably, transitions to a new subclone. Mutational signatures provide a unique way to recover tumour evolution path, track activities of mutational processes, adjust the treatment strategy and detect changes in therapy response.

## Methods

TrackSig is designed to be applied to VAF frequency data from a single, heterogeneous tumour sample. The method consists of two stages. First, we sort SNVs by their estimated CCF that we estimate using their VAFs and a CNA reconstruction of the samples. Next, we infer a trajectory of the mutational signature activities over the estimated ordering of the SNVs. We estimated activity trajectory for each signature as a piece-wise constant function of the SNV ordering with a small number of changepoints. These stages are described in detail below. Note that TrackSig does not rely on any methods for clustering mutations, such as phylogeny reconstruction.

### Ordering the SNVs

No single evolutionary model can yet explain all of the observed VAF distributions in bulk tumour samples^21,23,26,27,42^. Using a CCF-based ordering of SNVs allows TrackSig to track changes in signature activity under a variety of such models. The clonal evolution model of cancer^28^ posits that SNVs belong to one of a handful of subclones whose associated mutations all have the same CCF. Under this model, mutations from different subclones would occupy distinct regions of the CCF space, so if signature activities differ between subclones, the segments detected by TrackSig in CCF space would mark the presence of distinct subclones. Current neutral evolution models^21,26^ assume SNVs to be unique and persistent (i.e. the infinite sites assumption) and SNVs with higher CCFs generally occur earlier in the tumour’s evolution. Therefore, when SNVs are sorted in order of decreasing CCFs, TrackSig’s trajectories track changes in signature activity over time.

In the following sections, we describe how the SNV VAFs are used to create a timeline to which TrackSig is applied. For ease of presentation, we will assume that the time of SNV occurrence increases approximately monotonically with position on the timeline. This interpretation is valid under the infinite sites assumption and either a neutral evolution model or a clonal one when all subclones are from the same branch, as is often the case in single samples^31^. TrackSig’s reconstruction accuracy is tested on simulated data that is consistent with different evolutionary models and violations to these assumptions; and in the “Discussion” section, we discuss how to interpret TrackSig’s reconstructions when the timeline is not a faithful representation of time of SNV acquisition.

### Estimating CCF

Estimating a SNV’s CCF requires both an estimate of its VAF and an estimate of the average number of mutant and reference alleles per cell at the locus where the SNV occurs. In TrackSig, we derive this estimate from a CNA reconstruction provided with the VAF inputs.

To account for uncertainty in a SNV’s VAF due to the finite sampling, we model the posterior distribution over its VAF using a Beta distribution:

1 $${{{\rm{V}}}}{{{\rm{A}}}}{{{\rm{F}}}} \sim {{{\rm{Beta}}}}({n}_{{{{\rm{var}}}}},{n}_{{{{\rm{ref}}}}}),$$

where *n*_var_ is the number of reads carrying a variant, and *n*_ref_ is the number of reference reads. To simplify the algorithm, and the subsequent sorting step, we sample an estimate of VAF_*i*_ (VAF of SNV *i*) from this distribution and use that sample as a surrogate for the distribution in subsequent calculations. An advantage of this approach is that it gives us a single ordering. With a large number of SNVs, we expect little variability in the estimated activity trajectory due to uncertainty in the VAFs of individual SNVs. With a smaller number of SNVs, multiple orderings can be sampled and the trajectories combined.

If no CNA reconstruction is available, TrackSig assumes that each SNV is in a region of normal copy number and TrackSig estimates CCFs in autosomal regions by setting:

2 $${{{{\rm{CCF}}}}}_{i}=\frac{2\,\times\,{{{\rm{V}}}}{{{\rm{A}}}}{{{{\rm{F}}}}}_{i}}{{{{\rm{Purity}}}}},$$

where Purity is the purity (i.e. proportion of cancerous cells) of the sample. If purity is not provided, TrackSig assumes Purity = 1.

If a CNA reconstruction is available, TrackSig uses it when converting from VAF to CCF. TrackSig assumes there is a maximum of one copy of the variant allele per cell, and thus estimates CCF by setting:

3 $${{{{\rm{CCF}}}}}_{i}=\frac{(2+{{{\rm{Purity}}}}\,\times\, ({{{{\rm{CN}}}}}_{i}-2))}{{{{\rm{Purity}}}}}\,\times\, {{{\rm{V}}}}{{{\rm{A}}}}{{{{\rm{F}}}}}_{i},$$

where Purity is the purity of the sample, and ${{{{\rm{CN}}}}}_{i}$ is the clonal copy number of the locus. If the clonal CNA increases the number of variant alleles per cell, this will lead to CCFs larger than one. As such, these cases are easily detected and corrected. Specifically, if the observed VAF is >50% due to finite sampling noise, or there is more than variant allele per cell, the CCF_*i*_ calculated above could be >100% which cannot be correct. As such, in most cases, we will set CCF_*i*_ to be the minimum of Eq. (3) and 100%. When we do not do so, we will sometimes refer to these estimates as (estimated) “average number of mutant alleles per cell” to avoid confusion.

In regions of subclonal CNAs, estimating CCF requires a phylogenetic reconstruction in order to determine whether the subclonal CNA influences the number of variant alleles in the affected cells^22,43^. As such, when computing SNV ordering, by default, TrackSig filters SNVs in these regions out in order to avoid this time consuming operation. However, TrackSig can include these SNVs if provided with CCF estimates for them from methods that do consider subclonal CNAs^22,43,44^.

TrackSig sorts SNVs in order of decreasing estimated CCF and uses the rank of the SNV in this list as a "pseudo-time” estimate of its time of appearance. Note that this estimate will have a non-linear relationship to real time, if the overall mutation rate can vary during the tumour’s development. If some of the SNVs can be interpreted as clock mutations, an SNV’s rank can be converted into an estimate of real time^9^.

### Constructing a timeline

To derive an estimate of the activity trajectory, TrackSig converts the SNV ordering into a set of time points with non-overlapping subsets of the SNVs. We do this for two reasons. First, stable estimation of signature activities requires a minimum number of mutations. By binning mutations into time points and requiring a minimum number of time points per segment, TrackSig enforces a minimum of 100 mutations per segment. Also, the time complexity of TrackSig scales with the number of time points. So by binning mutations, we can speed up TrackSig. By default, we set the bin size to 100 but the user can change this setting to as low as 1. As we show in “Results” section, TrackSig’s signature activity reconstructions are relatively insensitive to the choice of bin size.

TrackSig first partitions the ordered mutations into bins and interprets each bin as one time point. The “timeline” of the cancer is the collection of the time points. TrackSig reports signature activity trajectories as a function of points in the timeline. We emphasize that TrackSig does not use any information about subclones when partitioning the SNVs and that TrackSig only uses CCFs for the SNV from a single sample.

### Computing activities of mutational signatures

To estimate activity trajectories, TrackSig partitions the timelines into segments containing one or more time points. Within each of these segments, it estimates signature activities using mixture of discrete distributions. Full details of the model are provided in the Supplementary Note 1. In brief, TrackSig models each signature as a discrete distribution over the *K* types and it treats the mutation count vector over the *K* types as a set of independently and identically distributed samples from a mixture of the discrete distributions corresponding to each signature. By default, TrackSig uses single-base tri-nucleotide signatures^2^ and *K* = 96, however TrackSig can use any mutation type labelling scheme, so long as it is given appropriate signatures as input. The mixing coefficients of these distributions are interpreted as their activities for the mixture model that produced the set of mutations. TrackSig fits these activities using the expectation-maximization algorithm (EM)^45^, as done by other signature activity estimation methods^30^.

### Detecting changepoints

TrackSig identifies changepoints in the timeline where there are discernible differences in the activity of mutations in the time points before and after the changepoints. Specifically, the changepoints partition the timeline into segments of mutations with approximately constant activities. TrackSig fits activities for this set using EM algorithm as described above. This procedure generates piece-wise constant activity trajectories for each signature. To select changepoints, we adapt pruned exact linear time (PELT)^46^, an optimal segmentation algorithm based on dynamic programming. We impose a complexity penalty at each time point that is equivalent to optimizing the Bayesian Information Criteria (BIC) (see Supplementary Note 2 for details). To reduce variance in our estimates of the signature activities, we do not allow partitions to be <100 mutations.

We compute the BIC criteria the following way. Changepoints split the timeline into (*#* changepoints + 1) segments. In each segment, TrackSig fits the signature activities, which have to sum to one. Therefore there are $(\#\ {{{\rm{signatures}}}}-1)$ free parameters per segment, or $(\#\ {{{\rm{changepoints}}}}+1)\cdot (\#\ {{{\rm{signatures}}}}-1)$ free parameters in total. As such, BIC objective takes the following form:

4 $${{{\rm{BIC}}}}=-2ln\hat{L}+(\#\ {{{\rm{changepoints}}}}+1)\cdot (\#\ {{{\rm{signatures}}}}-1)\cdot ln(\#\ {{{\rm{timepoints}}}}),$$

where $\hat{L}$ is the likelihood of the current model.

### Correcting the timeline and segment count

If the number of variant alleles per cell is increased by a clonal copy number change, TrackSig’s CCF estimates might be >1. To correct for this, when displaying activity trajectories, it merges all the time points that have average CCF ≥ 1 into one time point. As such, the first time point can contain more than 100 mutations. To determine a signature activity at this new time point, TrackSig simply takes an average activity of all merged time points (those having CCF ≥ 1).

To compute the number of distinct subclones, we adjust the number of detected changepoints to correct for overlap in the CCF space of mutations from different subclones. Consider the case of two subclones whose mutations overlap substantially in CCF space. In this case, TrackSig might find three segments instead of two: one with signatures activities reflecting the first subclone; another with activities reflecting a mixture of the two subclones; and last with activities reflecting the second subclone. If this happens, then the direction of change of all signatures will be the same in the two changepoints. As such, when counting the number of distinct subclones, we treat each such pair of changepoints as one subclone boundary. Such a situation only occurs in 2.6% of 2552 PCAWG tumour samples to which we applied TrackSig; in 77% of those cases we remove a single changepoint.

### Bootstrapping to estimating activity uncertainty

TrackSig estimates uncertainty in the activity estimates by bootstrapping the mutations and refitting the activity trajectories. Specifically, it takes the random subset of *N* mutations by sampling uniformly with replacement from the *N* unfiltered SNVs in the sample under consideration. Using the pre-assigned CCF estimates, we sort the SNVs in decreasing order, as above, re-partition them into time points and recompute activity estimates. The trajectories obtained from bootstrapped mutation sets have the same number of time points, however the average CCF for each time point can change. We use these bootstrapped trajectories to compute uncertainty estimates for the sizes of activity changes.

### Choosing active signatures

Only a subset of signatures are active in a particular sample, and this subset is largely determined by a cancer type. For the analyses reported above, we use a set of active signatures provided by PCAWG^47^, which contains a list of active signatures per sample (on average, four per sample). Frequent active signatures for each cancer type are available from a variety of sources^2,47^. We highly recommend using either these cancer-specific active signatures or deriving sample-specific active signatures using one of the procedures described in this section. We strongly discourage using TrackSig with a full set of signatures on a single sample, as many of the signatures overlap considerably, which can cause signature activity estimation errors due to this collinearity.

Here we evaluate three different ways to select the active signatures, all supported by TrackSig. The first strategy, “all-sigs”, simply computes activity trajectories for all signatures. The second, “cancer-type-specific-sigs”, uses all signatures reported as active in the cancer type under consideration. The final strategy, “sample-specific-sigs”, first fits signature activities to the full set of mutation counts using an initial set of signatures, and sets the active signatures to be those with activities greater than a threshold (by default, 5%) in the initial fit. Then TrackSig computes activity trajectories only for the active signatures. In the following, we evaluate “sample-specific-sigs” when the initial set is “all-sigs”, however, we suspect this approach will also work well with “cancer-type-specific-sigs” as the initial set. We evaluate each strategy by comparing the active signatures selected by TrackSig with those reported by PCAWG-Signature group on the PCAWG tumour set^47^.

For “all-sigs”, we used all 48 signatures and we found on average, 44.7% of overall activity assigned by TrackSig is assigned to the active signatures selected by PCAWG-Signature group. Each incorrect signature gets 1.3% of activity on average. In other words, the incorrect activity is widely distributed among the signatures. Using “cancer-type-specific-sigs” improves the correspondence to 68.7% of the total activity on average. This strategy reduces the initial set of potentially active signatures from 48 down to 12 on average (ranging from 4 signatures in Lower Grade Glioma to 24 signatures in Liver Cancer). Here, we observe that signature 5 and 40 are the most prevalent among the incorrect signatures, having the average activity of 14% and 12.6%, respectively in the samples where they are supposed to be inactive. Finally, if we use the “sample-specific-sigs” strategy starting with “all-sigs” as the initial set, we exactly recover the active signatures reported by PCAWG-Signature group.

Fitting either per cancer or per sample signatures results in more activity mass to be on the correct signatures and speeds up the computations. Therefore, we recommend choosing one of these instead of using activities from the full set.

### Reporting summary

Further information on research design is available in the Nature Research Reporting Summary linked to this article.

## Supplementary information

Supplementary Information

Peer Review File

Reporting Summary

## Data Availability

Somatic and germline variant calls, mutational signatures, subclonal reconstructions, transcript abundance, splice calls and other core data generated by the ICGC/TCGA Pan-cancer Analysis of Whole Genomes Consortium is described here^25^ and available for download at https://dcc.icgc.org/releases/PCAWG. Additional information on accessing the data, including raw read files, can be found at https://docs.icgc.org/pcawg/data/. In accordance with the data access policies of the ICGC and TCGA projects, most molecular, clinical and specimen data are in an open tier which does not require access approval. To access potentially identification information, such as germline alleles and underlying sequencing data, researchers will need to apply to the TCGA Data Access Committee (DAC) via dbGaP (https://dbgap.ncbi.nlm.nih.gov/aa/wga.cgi?page=login) for access to the TCGA portion of the dataset, and to the ICGC Data Access Compliance Office (DACO; http://icgc.org/daco) for the ICGC portion. In addition, to access somatic SNVs derived from TCGA donors, researchers will also need to obtain dbGaP authorisation.

## Code availability

TrackSig Code is available at https://github.com/morrislab/TrackSig. Code for generating simulation data is included in the Github repository. The core computational pipelines used by the PCAWG Consortium for alignment, quality control and variant calling are available to the public at https://dockstore.org/search?search=pcawgunder the GNU General Public License v3.0, which allows for reuse and distribution.
