# Supplementary material for: Reconstructing evolutionary trajectories of mutation signature activities in cancer using TrackSig
Source: Nat Commun. 2020 Feb 5;11:731. doi: 10.1038/s41467-020-14352-7 (PMC7002414; doi:10.1038/s41467-020-14352-7)
Supplement: Supplementary file 3 — Reporting Summary [file 41467_2020_14352_MOESM3_ESM.pdf]

nature research

Corresponding author(s): Quaid Morris

Reporting Summary

Nature Research wishes to improve the reproducibility of the work that we publish. This form provides structure for consistency and transparency in reporting. For further information on Nature Research policies, see Authors & Referees and the Editorial Policy Checklist.

Statistical parameters

When statistical analyses are reported, confirm that the following items are present in the relevant location (e.g. figure legend, table legend, main text, or Methods section).

n/a	Confirmed	
☐	☒	The exact sample size (*n*) for each experimental group/condition, given as a discrete number and unit of measurement
☐	☒	An indication of whether measurements were taken from distinct samples or whether the same sample was measured repeatedly
☒	☐	The statistical test(s) used AND whether they are one- or two-sided<br>*Only common tests should be described solely by name; describe more complex techniques in the Methods section.*
☒	☐	A description of all covariates tested
☐	☒	A description of any assumptions or corrections, such as tests of normality and adjustment for multiple comparisons
☐	☒	A full description of the statistics including central tendency (e.g. means) or other basic estimates (e.g. regression coefficient) AND variation (e.g. standard deviation) or associated estimates of uncertainty (e.g. confidence intervals)
☒	☐	For null hypothesis testing, the test statistic (e.g. *F*, *t*, *r*) with confidence intervals, effect sizes, degrees of freedom and *P* value noted<br>*Give P values as exact values whenever suitable.*
☒	☐	For Bayesian analysis, information on the choice of priors and Markov chain Monte Carlo settings
☒	☐	For hierarchical and complex designs, identification of the appropriate level for tests and full reporting of outcomes
☒	☐	Estimates of effect sizes (e.g. Cohen's *d*, Pearson's *r*), indicating how they were calculated
☐	☒	Clearly defined error bars<br>*State explicitly what error bars represent (e.g. SD, SE, CI)*

Our web collection on statistics for biologists may be useful.

Software and code

Policy information about availability of computer code

Data collection	Data was collected and processed as a part of PanCancer Analysis of Whole Genomes initiatives. The PCAWG dataset will be released shortly and become publicly available though standard methods. The list of mutational signatures used for PCAWG analysis will be released at the same time.
Data analysis	Our paper presents the new method for analyzing cancer data. The code is available in the open-source repository: https://github.com/YuliaRubanova/TrackSig. The repository include the code of TrackSig method, example of the input data and code for generating simulations.

For manuscripts utilizing custom algorithms or software that are central to the research but not yet described in published literature, software must be made available to editors/reviewers upon request. We strongly encourage code deposition in a community repository (e.g. GitHub). See the Nature Research guidelines for submitting code & software for further information.

Data

Policy information about availability of data

All manuscripts must include a data availability statement. This statement should provide the following information, where applicable:

- Accession codes, unique identifiers, or web links for publicly available datasets
- A list of figures that have associated raw data
- A description of any restrictions on data availability

We have used simulated data as well as the data from PanCancer Analysis of Whole Genomes (PCAWG) study. The code for generating simulated data is provided on GitHub: https://github.com/YuliaRubanova/TrackSig. The PCAWG dataset and PCAWG mutational signatures will be released shortly and become publicly available though standard methods. Currently the PCAWG data is partially available on ICGC website: https://dcc.icgc.org/releases/PCAWG/consensus_snv_indel.

Below is the list of datasets used to generate the figures and tables:
PCAWG: Figures 1-4, figure C.1
Simulated data: Figures 5-6, tables 1-2

Field-specific reporting

Please select the best fit for your research. If you are not sure, read the appropriate sections before making your selection.

☒ Life sciences ☐ Behavioural & social sciences ☐ Ecological, evolutionary & environmental sciences

For a reference copy of the document with all sections, see nature.com/authors/policies/ReportingSummary-flat.pdf

Life sciences study design

All studies must disclose on these points even when the disclosure is negative.

Sample size	PCAWG dataset: not applicable as the dataset was collected prior to our work.<br>Simulated data: we have created a set simulations with different combinations of four mutational signatures with 1, 2 or 3 change-points or no change-points (4140 simulations in total). In comparison, real cancer samples contain 4 signatures and less than 1 change-points on average per tumour.
Data exclusions	We exclude the cancer samples with less than 600 SNVs. Cancer samples with less than 600 SNVs have less than timeline of less than 6 time points, which is insufficient for performing the change-point finding algorithm.
Replication	TrackSig algorithm is deterministic, therefore, given the same mutation counts, the results are guaranteed to be reproducible. For every cancer sample or simulations, we bootstrap the data and re-run the analysis 30 times to ensure the accurate signature activity reconstruction and change-point detection. On PCAWG data, we observe the mean standard deviation of activity values to be 2.9%. Note that the simulations in TrackSig paper were generated using PCAWG mutational signatures, which are now released. In the github repository we include only publicly available mutational signatures from COSMIC: https://cancer.sanger.ac.uk/cosmic/signatures.
Randomization	PCAWG dataset: not applicable as the dataset was collected prior to our work.<br>Simulated data: not applicable as the simulations do not contain any patient data.
Blinding	PCAWG dataset: not applicable as the dataset was collected prior to our work.<br>Simulated data: not applicable as the simulations do not contain any patient data.

Reporting for specific materials, systems and methods

Materials & experimental systems

n/a	Involved in the study
☒	☐ Unique biological materials
☒	☐ Antibodies
☒	☐ Eukaryotic cell lines
☒	☐ Palaeontology
☒	☐ Animals and other organisms
☒	☐ Human research participants

Methods

n/a	Involved in the study
☒	☐ ChIP-seq
☒	☐ Flow cytometry
☒	☐ MRI-based neuroimaging
